# A reciprocal feedback between N6-methyladenosine reader YTHDF3 and lncRNA DICER1-AS1 promotes glycolysis of pancreatic cancer through inhibiting maturation of miR-5586-5p

**DOI:** 10.1186/s13046-022-02285-6

**Published:** 2022-02-19

**Authors:** Yuhang Hu, Jiang Tang, Fengyu Xu, Jinhuang Chen, Zhu Zeng, Shengbo Han, Fan Wang, Decai Wang, Mengqi Huang, Yong Zhao, Yan Huang, Wenfeng Zhuo, Gang Zhao

**Affiliations:** Department of Emergency Surgery, Union Hospital, Tongji Medical College, Huazhong University of Science and Technology, Wuhan, 430022 China

**Keywords:** LncRNA, DICER1-AS1, DICER1, Pancreatic cancer, Glycolysis, m6A, YTHDF3, miR-5586-5p

## Abstract

**Background:**

Glycolysis is a pivotal process in metabolic reprogramming of tumorigenesis. Previous research has indicated that lncRNAs might play crucial roles in glycolysis of various tumors. However, the function of lncRNAs in glycolysis of pancreatic cancer has not been fully elucidated.

**Methods:**

Bio-information analyses were applied to reveal the potential glycolysis-associated lncRNA. RT-PCR and fluorescence in situ hybridization (FISH) assays were applied to detect the expression of antisense RNA1 of DICER1 (DICER1-AS1) in pancreatic cancer tissues and cell lines. Gain- and loss-of-function experiments were performed to evaluate the roles of DICER1-AS1 in glycolysis and tumorigenesis of PC. Mechanistic experiments including luciferase reporter assay, RNA immunoprecipitation (RIP), and chromatin immunoprecipitation (ChIP) were employed to uncover the downstream targets and regulatory mechanism of DICER1-AS1 in glycolysis of PC.

**Results:**

Bio-information analysis indicated that DICER1-AS1 was downregulated in PC and negatively correlated with glycolytic genes expression. Meanwhile, overexpression of DICER1-AS1 inhibited glycolysis, proliferation, and metastasis of PC cells both in vitro and in vivo. Mechanistically, DICER1-AS1 promoted transcription of its sense gene DICER1 by recruiting transcriptional factor YY1 to the DICER1 promoter. Meanwhile, DICER1 promoted maturation of miR-5586-5p which consequently inhibited glycolytic gene expression including LDHA, HK2, PGK1, and SLC2A1. Notably, enhanced interaction between N6-methyladenosine (m6A) reader YTHDF3 and DICER1-AS1 led to degradation of DICER1-AS1 in response to glucose depletion. Moreover, our data revealed that YTHDF3 was a critical target for miR-5586-5p, by which forming a negative feedback with DICER1-AS1 to regulate glycolysis of PC.

**Conclusion:**

Our results implicate a negative feedback of m6A reader YTHDF3 and glycolytic lncRNA DICER1-AS1 is involved in glycolysis and tumorigenesis of PC.

**Supplementary Information:**

The online version contains supplementary material available at 10.1186/s13046-022-02285-6.

## Background

To maintain the urgent demand for tumorigenesis and aggressiveness, pancreatic cancer cells have extensively reprogrammed energy metabolism [1]. One of the most common and important alterations of metabolism is aerobic glycolysis, which is termed the “Warburg effect”. Glycolysis displays a less efficient metabolism with more lactate production, lower extracellular pH value, and more glucose consumption, even under an oxygen-enriched microenvironment [2]. Pancreatic cancer cells show significantly activated glycolysis, which was associated with poor prognosis in pancreatic cancer (PC) patients [3]. Thereby, targeting the Warburg effect has been regarded as a promising therapeutic direction in tumors. Recent studies revealed that disrupting SLC2A1, LDHA or other key glycolytic enzymes resulted in remarkably reduced conversion of glucose to pyruvate and ATP, and elevated mitochondrial oxidative phosphorylation [4]. Meanwhile, inhibitors of SLC2A1 or LDHA in combination with tumor chemotherapeutics displayed significant synergistic antitumor effect in vitro, which highlighting the promising role for cancer therapeutic strategy of glycolysis [5, 6]. Nevertheless, the metabolic process and molecular mechanisms of glycolysis in PC progression remain uncertain and need to be further determined.

Long noncoding RNAs (lncRNAs) are a type of special transcript longer than 200 nucleotides and lack protein-coding potential. Although lncRNAs were regarded as merely transcriptional ‘‘noise’’ before, emerging evidence has shown that lncRNAs play pivotal roles in transcriptional regulation, epigenetic gene regulation, and tumors [7]. And our previous study has revealed that lncRNA GLS-AS, dysregulated under glucose and glutamine deprivation, repressed pancreatic cancer progression by repressing the Myc/GLS pathway [8]. Specifically, emerging studies have shown the key role of lncRNAs in the regulation of glycolysis in cancers. For example, lncRNA MACC1-AS1 promotes glycolysis progression and anti-oxidative capabilities under metabolic stress in gastric cancer [9]. Besides, lncRNA LINRIS has been reported as an independent prognostic factor that participated in the regulation of aerobic glycolysis of colorectal cancer [10]. Similarly, Wang et al. have reported that lncRNA PVT1 facilitates PC glycolysis and progression by regulating the miR-519d-3p/HIF-1A pathway [11]. Nevertheless, the molecular mechanisms of lncRNAs for the Warburg effect and its role in PC progression have not been fully discovered. Therefore, our present study sought to find a lncRNA that is critical for glycolysis and tumorigenesis of PC.

In this study, we identified that DICER1-AS1 was down-regulated in PC and was correlated with a poor prognosis. Meanwhile, both in vitro and in vivo experiments validated that DICER1-AS1 functioned as an inhibitor of PC in glycolysis and tumorigenesis by promoting DICER1-mediated maturation of miR-5586-5p. Notably, we further disclosed DICER1-AS1 was degraded in glucose deprivation and further explored the m6A-correlated regulation.

## Methods

### Cell culture

The cell lines (SW1990, AsPC-1, PANC-1, BxPC-3) of human pancreatic cancer were obtained from the American Type Culture Collection (ATCC, USA). The normal pancreatic ductal epithelial cell line (HPDE) was obtained from Bena Culture Collection (BNCC, China). Details are provided in Additional file 1.

### Patients and clinical samples

The clinical samples including tumor tissues and matched noncancerous tissues were obtained from patients with pancreatic cancer at the Pancreatic Disease Institute of Union Hospital (Wuhan, China). Details are provided in Additional file 1.

### RNA pull-down assay

For RNA pull-down assay, RNA was synthesized in vitro via RT-PCR using specific primers containing a T7 RNA-polymerase promoter sequence according to the manufacturer's instructions of MAXIscript® Kit (Ambion, USA). Then the RNA was end-labeled with desthiobiotin by using Pierce RNA 3′ End Desthiobiotinylation Kit (Thermo Scientific, USA). Finally, RNA pull-down assay was performed using the Pierce™ Magnetic RNA–Protein Pull-Down Kit (Thermo Scientific, USA) according to the manufacturer's instructions. Details are provided in Additional file 1.

### MeRIP

m6A modifications of RNAs were measured by methylated RNA immunoprecipitation (MeRIP) assay. Details are provided in Additional file 1.

### Statistical analysis

All statistical analyses were performed using R-3.0.2 software (http://cran.r-project.org/bin/windows/base/old/3.0.2/). Details are provided in Additional file 1.

### Further applied methods

Additional RNA isolation, reverse transcription, and quantitative real-time PCR (qRT-PCR), cell transfection, RNA fluorescence in situ hybridization (RNA-FISH) assay, MTT assay, Wound healing assay, Transwell assay, Western blot assay, xenograft assay, Glucose uptake assay, Lactate Production Assay, Luciferase reporter assay, RNA stability assay, Chromatin Immunoprecipitation (ChIP) assay, RNA pull-down assay, RNA-binding protein immunoprecipitation (RIP), immunohistochemistry (IHC), Immunoflourescence, Gene set enrichment analysis (GSEA) are further described in the Additional file 1.

## Results

### DICER1-AS1 is downregulated in pancreatic cancer tissues and negatively correlated with glycolysis pathway

**Fig. 1:**
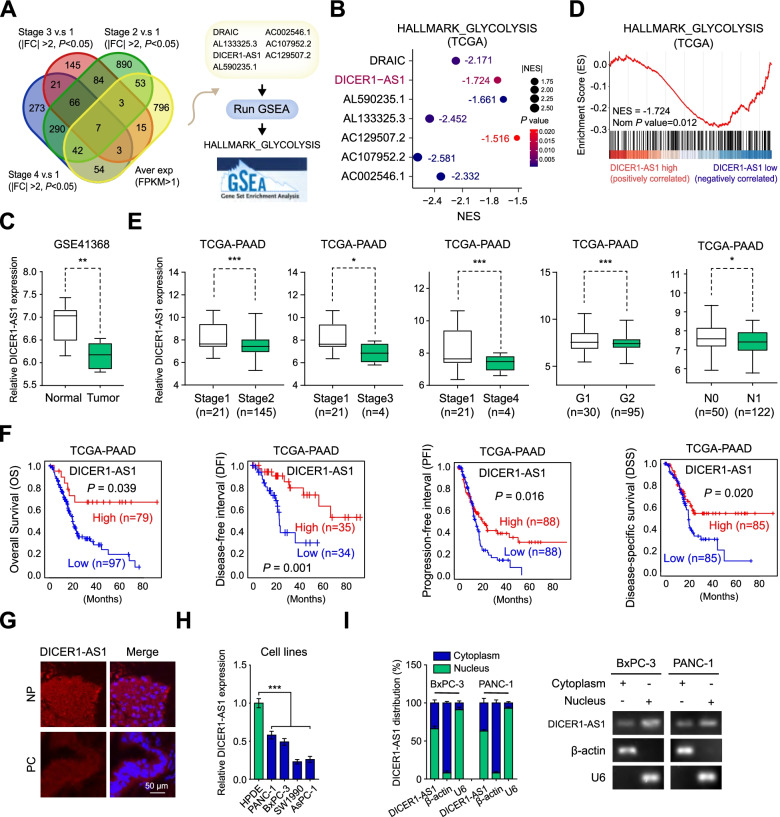
DICER1-AS1 is downregulated in pancreatic cancer tissues and negatively correlated with glycolysis pathway.** A** Venn diagram (left panel) indicating the identification of differentially expressed lncRNAs (|log2FoldChange|> 1, *P* < 0.05) in the TCGA database. The right panel showing the identification of glycolysis-related lncRNAs by GSEA analyses. **B** Bubble plot showing the GSEA analyses of 7 lncRNAs using a glycolysis-related gene set in the TCGA database. NES, normalized enrichment score. **C** Boxplot showing the relative level of DICER1-AS1 between normal and tumor tissues in pancreatic cancer (GSE41368). **D** GSEA enrichment plot of DICER1-AS1 using glycolysis-related gene set in the TCGA database. NES, normalized enrichment score. **E** The relative transcript levels of DICER1-AS1 in PC tissues with different status of tumor stage and progression. **F** Kaplan–Meier analyses of overall survival (OS) (cutoff value = 193.92), disease-free interval (DFI) (cutoff value = 190.87), progression-free interval (PFI) (cutoff value = 177.20), and disease-specific survival (DSS) (cutoff value = 177.20) in pancreatic cancer patients with low and high levels of DICER1-AS1 using the log-rank test. **G** Representative FISH images of DICER1-AS1 expression in adjacent noncancerous pancreatic (NP) tissues and pancreatic cancer (PC) tissues. Blue, DAPI staining; Red, DICER1-AS1 staining; Scale bar = 20 µm. **H** Real-time qRT-PCR showing the relative DICER1-AS1 levels in a normal pancreatic cell line (HPDE) and PC cell lines (SW1990, AsPC-1, PANC-1, BxPC-3). **I** Real-time qRT-PCR showing in the subcellular fractions of DICER1-AS1 (left panel) in BxPC-3 and PANC-1 cells (mean ± SD, n = 3). The PCR products were run on a 2% agarose gel and U6 and β-actin were used as nuclear and cytoplasmic markers, respectively (right panel). All data were presented as means ± SD of at least three independent experiments. Values are significant at *P < 0.05, **P < 0.01 and ***P < 0.001 as indicated

To explore the potential glycolysis-correlated lncRNAs in pancreatic cancer, we first analyzed the public dataset of pancreatic adenocarcinoma (PAAD) derived from The Cancer Genome Atlas (TCGA) database [12]. We identified 7 lncRNAs that were differentially expressed in PC patients based on the varied clinical progression, and negatively associated with the glycolysis pathway by the analysis from the gene set enrichment analysis (GSEA) [13] (Fig. 1A-B, Additional file 3: Figure S1A). Among these lncRNAs, antisense RNA of DICER1 (DICER1-AS1) was downregulated in PC tissues derived from GSE41368 dataset (Fig. 1C), then was specifically focused. The GSEA analysis demonstrated that DICER1-AS1 was negatively correlated with gene sets including glycolysis (Fig. 1D), cell metastasis, and pancreatic cancer (Additional file 3: Figure S1B). Gene ontology (GO) analyses revealed that DICER1-AS1 was correlated carbohydrate catabolic process and production of miRNA (Additional file 3: Figure S1C). Coincidentally, GSEA analysis based on GSE41368 also revealed that DICER1-AS1 was negatively correlated with glycolysis process (Additional file 3: Figure S1D). The TCGA database revealed that DICER1-AS1 was downregulated in PC with advanced clinical stages and tumor grade, and correlated with longer overall survival (OS), disease-free interval (DFI), progression-free interval (PFI), and disease-specific survival (DSS) (Fig. 1F, Additional file 3: Figure S1A). Meanwhile, DICER1-AS1 level was also downregulated in PC tissues derived from cohort 1 (Additional file 2: Table S3), which was composed of 86 paired PC tissues and normal tissues, as well as correlated with longer overall survival (Additional file 3: Figure S1E-F). Furthermore, receiver operating characteristic (ROC) curves showed that DICER1-AS1 can identify PC patients from healthy individuals both in the GEO database (GSE41368, GSE16515) and cohort 1 (Additional file 3: Figure S1G). Subsequently, we analyzed the correlation of DICER1-AS1 expression with different clinicopathological features in our cohort1 and found that the DICER1-AS1 level was significantly associated with tumor size and TNM stage (Additional file 3: Figure S1H). Univariate and multivariate regression analyses showed that DICER1-AS1 expression was an independent predictor of PC patients with significant hazard ratios for predicting clinical outcomes (Additional file 2: Table S4).

Meanwhile, FISH analysis confirmed that DICER1-AS1 expression was lower in PC tissues compared with normal pancreatic (NP) tissues (Fig. 1G). Besides, DICER1-AS1 expression was significantly lower in different pancreatic cell lines than normal human pancreatic duct epithelial cells (HPDE) (Fig. 1H). For a good efficiency of RNA interference experiments, further study was investigated in PC cells (PANC-1, BxPC-3) with relatively high expression of DICER1-AS1. Subcellular fractionation assays showed that DICER1-AS1 is located both in the nucleus and cytoplasm (Fig. 1H). Furthermore, the online coding potential tool (CPC and CPAT) [14, 15] confirmed that DICER1-AS1 has a very low protein-coding ability (Additional file 3: Figure S2A). Besides, ribosome profiling data showed a remarkably low-level peak in the DICER1-AS1 gene indicating a low protein-coding ability of DICER1-AS1 [16] (Additional file 3: Figure S2B). Meanwhile, we found that DICER1-AS1 has low evolutionary conservation by using PhyloP analysis across 100 vertebrates (Additional file 3: Figure S2C). After mining public datasets (32 studies) of cBioPortal for Cancer Genomics, we found that there was no amplification and deep deletion of the DICER1-AS1 gene in pancreatic cancer (Additional file 3: Figure S2D). Taken together, these results implied that DICER1-AS1 was a glycolysis-associated lncRNA that might function as an inhibitor in PC progression.

### DICER1-AS1 inhibits glycolysis, proliferation and metastasis of PC cells

**Fig. 2:**
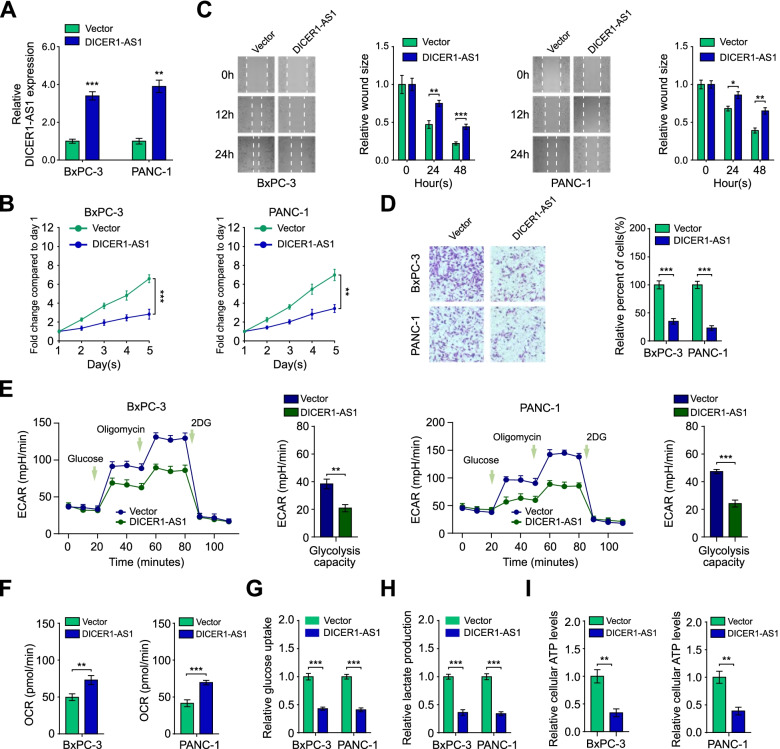
DICER1-AS1 inhibits glycolysis, proliferation and metastasis of PC cells. A Real-time PCR analysis of DICER1-AS1 level in BxPC-3 and PANC-1 cells after transfected with the pcDNA-DICER1-AS1 plasmid (DICER1-AS1) and negative control (Vector), respectively. **B** The proliferative capability was performed by MTT assay in BxPC-3 and PANC-1 cells transfected with pcDNA-DICER1-AS1 (DICER1-AS1) or empty vector control (Vector). **C** Migration ability was assessed by wound healing assay in BxPC-3 and PANC-1 cells transfected with pcDNA-DICER1-AS1 (DICER1-AS1) or empty vector control (Vector). **D** Transwell assay was applied to assess the invasion ability of those cells transfected indicated above. **E** The change of ECAR levels in BxPC-3 and PANC-1 cells transfected with vector or DICER1-AS1 (*n* = 3). The glucose (10 mmol·L^−1^), oligomycin (2 μmol·L^−1^), or 2-deoxyglucose (2-DG, 50 mmol·L^−1^) were used at indicated points. **F** Seahorse extracellular flux assay showing the oxygen consumption rate (OCR) in BxPC-3 and PANC-1 cells transfected with vector or DICER1-AS1 (*n* = 3). **G** The glucose uptake levels in BxPC-3 and PANC-1 cells transfected with control or DICER1-AS1 vectors (*n* = 3). **H** The relative lactic acid levels were detected in BxPC-3 and PANC-1 cells transfected with control or DICER1-AS1 vectors (*n* = 3). **I** The ATP levels in BxPC-3 and PANC-1 cells transfected with control or DICER1-AS1 vectors (*n* = 3). All data were presented as means ± SD of at least three independent experiments. Values are significant at ^a^*P* < 0.05, ^b^P < 0.01 and ^c^P < 0.001 as indicated

To further investigate the biological impact of DICER1-AS1 in pancreatic cancer cells, DICER1-AS1 was manipulated with overexpressed plasmid or siRNA (siDICER1-AS1#1–3), respectively (Fig. 2A, Additional file 3: Figure S3A). We found that the overexpression of DICER1-AS1 significantly inhibited (Fig. 2B-D), while the knockdown of DICER1-AS1 obviously increased the proliferation, migration, and invasion of BxPC-3 and PANC-1 cells (Figure S3B-D). We next identified whether altered DICER1-AS1 levels directly influence glycolytic metabolism in pancreatic cancer cells. Indeed, overexpression of DICER1-AS1 significantly decreased, while DICER1-AS1 knockdown increased the extracellular acidification rate (ECAR), glucose consumption, lactate production, and ATP level. On the contrary, oxygen consumption rate (OCR) was notably increased by DICER1-AS1 overexpression but decreased by DICER1-AS1 knockdown (Fig. 2E-I, Additional file 3: Figure S3E-I). In addition, the 2-DG, an inhibitor of glycolysis, significantly blocked the glucose uptake and lactate production induced by DICER-AS1 knockdown (Additional file 3: Figure S3J-K). These data indicate that DICER1-AS1 is an inhibitor for pancreatic cancer by suppressing glycolysis.

### DICER1 is a critical target for DICER1-AS1 regulating glycolysis

**Fig. 3:**
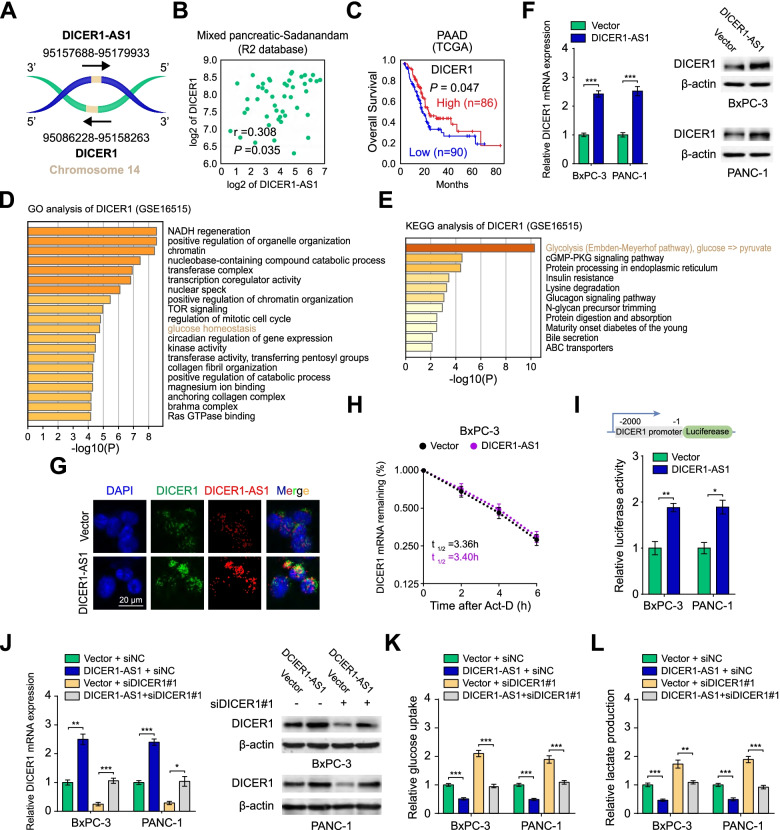
DICER1 is a critical target for DICER1-AS1 regulating glycolysis. A Schematic illustration showing the genomic location of DICER1-AS1 and DICER1. **B** The expression correlation between DICER1-AS1 and DICER1 in PC tissues derived from the R2 database (https://hgserver1.amc.nl/cgi-bin/r2/main.cgi). **C** Kaplan–Meier survival analysis of PC patients with high or low levels of DICER1 (cutoff value = 2886.20) derived from the TCGA database. **D**-**E** GO and KEGG analyses of DICER1-correlated genes in PC tissues from GEO database (GSE16515). **F** Real-time qRT-PCR assay (left panel) and western blot assay (right panel) showing the expression of DICER1 in BxPC-3 and PANC-1 cells transfected with control or DICER1-AS1 plasmids. (n = 3). **G** RNA-FISH and immunofluorescence detection of DICER1-AS1 (red) and DICER1 (green) in DICER1-AS1 overexpressed cells (BxPC-3). **H** The DICER1 mRNA half-life (t_1/2_) was detected by real-time PCR in BxPC-3 cells transfected with control vector or DICER1-AS1 plasmids. **I** Schematic illustration of luciferase reporter constructs used for examining the effects of DICER1-AS1 on the DICER1 promoter (upper panel). The dual-luciferase assay showing the relative activity of DICER1 promoter in BxPC-3 and PANC-1 cells transfected with control vector or DICER1-AS1 (lower panel). **J** Real-time qRT-PCR (left panel) and western blot assay (right panel) showing the change of transcript and protein levels of DICER1 in BxPC-3 and PANC-1 cells transfected with control or DICER1-AS1 plasmids and those co-transfected with siNC or siDICER1. **K**-**L** The relative glucose uptake levels and lactic acid production were detected in BxPC-3 and PANC-1 cells transfected with control or DICER1-AS1 plasmids and those co-transfected with siNC or siDICER1. All data were presented as means ± SD of at least three independent experiments. Values are significant at ^a^P < 0.05, ^b^P < 0.01 and ^c^P < 0.001 as indicated

Since DICER1-AS1 is an antisense RNA of DICER1 (Fig. 3A), we wonder whether DICER1 might be a critical target of DICER1-AS1. Coincidently, DICER1-AS1 was positively correlated with DICER1 expression in pancreatic cancer (Fig. 3B), as well as in a variety of cancers or normal tissues (Additional file 3: Figure S4A and 4B). Moreover, TCGA and GEO databases displayed that low expression of DICER1 correlated with a poor overall survival both in pancreatic cancer (Fig. 3C) and a variety of malignant tumors (Additional file 3: Figure S4C). Meanwhile, the copy number of the DICER1 gene was neither significantly altered in the different clinical stages nor associated with the survival of PC patients from the TCGA database (Additional file 3: Figure S5A-B), which indicates that the mRNA level but not copy number of DICER1 gene is decisive for pancreatic cancer.

Coincidently, GO and Kyoto Encyclopedia of Genes and Genomes (KEGG) [17] analyses further indicated that DICER1 was related to glucose homeostasis and glycolysis (Fig. 3D-E). Meanwhile, overexpression or knockdown of DICER1-AS1 could increase or decrease DICER1 expression of PC cells in both mRNA and protein level (Fig. 3F, Additional file 3: Figure S5C-D), which was further validated with RNA-FISH and immunofluorescence assays (Fig. 3G, Additional file 3: Figure S5E). After being treated with transcription inhibitor actinomycin D, the decay of DICER1 was not affected by overexpression or depletion of DICER1-AS1 both in BxPC-3 and PANC-1 cells (Fig. 3H, Figure S5F), which indicated that DICER1 would be regulated by DICER1-AS1 in transcription but not post-transcription level. Additionally, DNA fragments containing the DICER1 promoter were inserted into the promoter region of firefly luciferase reporter plasmids to perform the luciferase assay. The results showed that overexpression of DICER1-AS1 enhanced, while depletion of DICER1-AS1 decreased the transcriptional activity of the DICER1 promoter (Fig. 3I, Figure S5G). These results further confirmed that DICER1-AS1 regulated DICER1 expression at the transcriptional level.

To further investigate the importance of DICER1 in the regulation of glycolysis, DICER1-AS1/DICER1 pathway was modulated by rescue or reverse experiments. Results showed that overexpression or knockdown of DICER1-AS1 induced or inhibited the levels of DICER1, which could be reversed by inhibition or overexpression of DICER1 (Fig. 3J, Figure S5H). Moreover, depletion of DICER1 obviously rescued the glucose uptake and lactate production of PC cells which were inhibited by DICER1-AS1 overexpression (Fig. 3K-L). In contrast, the glucose uptake and lactate production promoted by DICER1-AS1 knockdown was remarkably reversed by DICER1 overexpression (Additional file 3: Figure S5I-J). Collectively, our data intensively implied that DICER1-AS1 regulates the glycolysis of PC cells by regulating DICER1 expression.

### Transcription factor YIN-YANG-1 (YY1) is a DICER1-AS1 binding protein

**Fig. 4:**
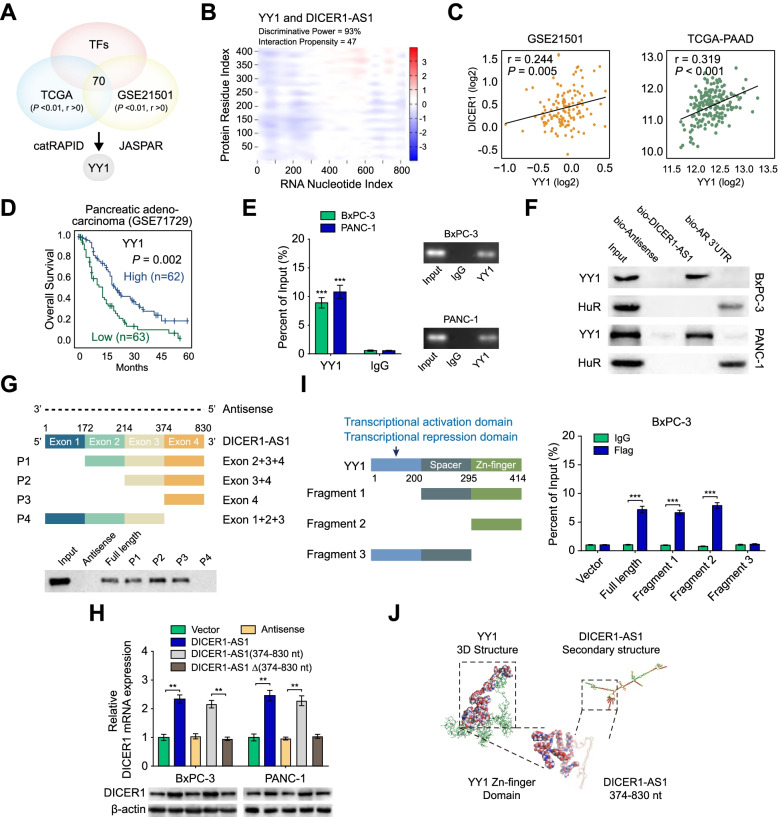
DICER1-AS1 binding protein YY1 is essential for the expression of DICER1. A The diagram for selected candidate transcription factors from the TCGA and GEO (GSE21501) database using the catRAPID and JASPAR website. **B** The heatmap showing the prediction of the interaction between DICER1-AS1 and YY1 protein. The colors of the heatmap showing the interaction score (ranging from -3 to 3) of the individual amino acid and nucleotide pairs. The interaction propensity between DICER1-AS1 and YY1 protein is 47 and the discriminative power is 93%. **C** The expression correlation between DICER1-AS1 and DICER1 in PC tissues derived from the GEO database (GSE21501) and the TCGA database. **D** Kaplan–Meier survival analysis of patients with high or low levels of YY1 in PC patients derived from the GEO database (cutoff value = 5.81). **E** RIP assay was applied using the anti-YY1 antibody and IgG antibody. The detection of DICER1-AS1 (left panel) was performed using specific primers in BxPC-3 and PANC-1 cells. Then the PCR products were run on a 2% agarose gel (right panel). **F** Western blot of the proteins from RNA pull-down assays using anti-YY1 and anti-HuR antibodies. RNA from the 3´ untranslated region (UTR) of the androgen receptor (AR) served as the control, containing UC-rich HuR binding regions. **G** Western blot of YY1 pulled down by full-length (F1: 1–830) or a series of DICER1-AS1 truncates or antisense in BxPC-3 cells. **H** DICER1 mRNA (upper panel) and protein (lower panel) were detected in BxPC-3 and PANC-1 cells transfected with empty vector, DICER1-AS1, antisense or DICER1-AS1 truncates. **I** RIP assay (right panel) was performed in BxPC-3 cells to detect the binding efficiencies between different fragments of YY1 protein with DICER1-AS1. Schematic structures of YY1 protein and three truncated mutants of YY1 variants were used in this study (left panel). **J** Visualization of interaction between the 3D structure of the YY1 zinc-finger domain and DICER1-AS1 (374–830 nt). All data were presented as means ± SD of at least three independent experiments. Values are significant at ^a^P < 0.05, ^b^P < 0.01 and ^c^P < 0.001 as indicated

To explore the underlying regulatory role of DICER1-AS1 on DICER1, we performed an intersection analysis of transcription factors which were correlated with DICER1 expression (*P* < 0.01, r > 0) in TCGA and GEO database. Further over-lapping analysis with protein-RNA pairs estimating database *catRAPID* [18] and transcription factor binding database *JASPAR* (https://jaspar.genereg.net/) revealed that transcription factor YY1 might participate in DICER1-AS1/DICER1 pathway (Fig. 4A). As shown in Fig. 4B, the catRAPID algorithm showed an intensive interaction between DICER1-AS1 and YY1. Meanwhile, analysis of RNA-seq data from the GEO, TCGA, and CCLE databases demonstrated that DICER1 was significantly correlated with YY1 mRNA expression both in cancer tissue and tumor cell lines (Fig. 4C, Additional file 3: Figure S6A). Moreover, low expression of YY1 was associated with poor outcomes of PC patients and other types of cancers derived from the TCGA and GEO database (Fig. 4D, Additional file 3: Figure S6B).

To verify the interaction between DICER1-AS1 and YY1, RIP assay was applied by using the anti-YY1 antibody and IgG antibody. The RIP assay confirmed a specific binding between DICER1-AS1 to YY1 both in BxPC-3 and PANC-1 cells (Fig. 4E). Furthermore, biotin-labeled RNA pull-down and western blot assays further validated the interaction of YY1 with DICER1-AS1 (Fig. 4F). Besides, deletion-mapping analyses indicated that exon 4 (374-830nt) of DICER1-AS1 was essential for its interaction with YY1 protein (Fig. 4G). Coincidently, overexpression of full or 374-830nt of DICER1-AS1, but not depletion of 374-830nt and antisense, could increase the expression of DICER1 in BxPC-3 and PANC-1 cells (Fig. 4H). To determine which functional domain of DICER1 is essential for the interaction with DICER1-AS1, RIP assay was performed by using Flag-tagged full-length and truncated mutants of YY1 protein. The results showed that the zinc-finger (295–414 aa) domain of Flag-tagged DICER1 protein was crucial for its interaction with DICER1-AS1 (Fig. 4I). The TASSER and SWISS-MODEL software [19, 20] were used to predict the 3D structure of the full-length and the zinc-finger domain of YY1 protein, respectively. And the RNA secondary structure of DICER1-AS1 was predicted via the RNA fold web server. Then the visual structure of the YY1/DICER1-AS1 binding complex was presented in Fig. 4J. Collectively, these results indicated that YY1 is a DICER1-AS1 binding protein which might be essential for DICER1 expression.

### DICER1-AS1 promotes DICER1 transcription by recruiting YY1 to the DICER1 promoter

**Fig. 5:**
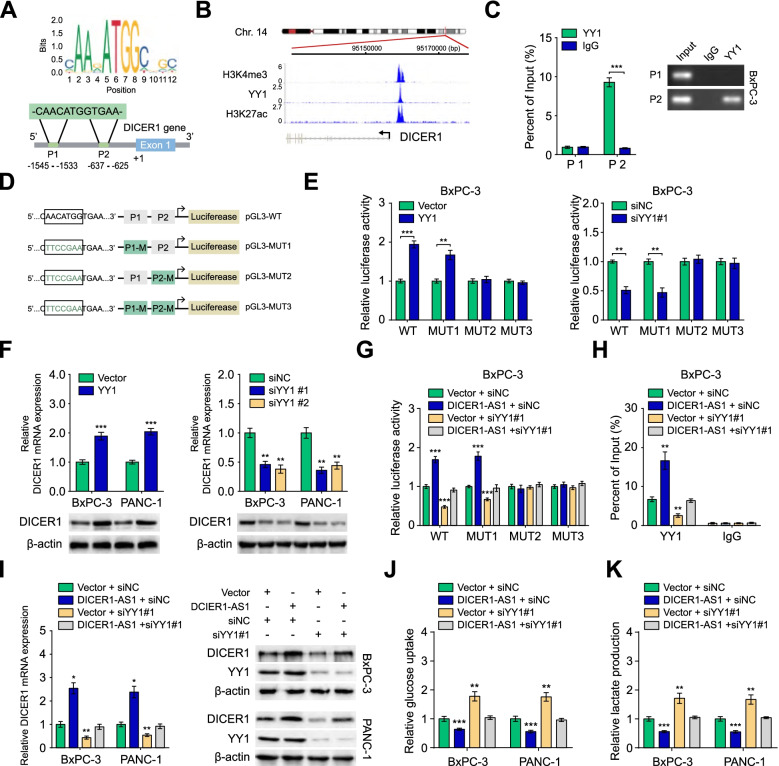
DICER1-AS1 promotes DICER1 transcription by recruiting YY1 to the promoter of DICER1. A Prediction of the consensus YY1 binding sequence was presented via the JASPAR database (upper panel). Schematic illustration showing the prediction of YY1 binding sites of DICER1 promoter via the JASPAR database (lower panel). **B** ChIP-Seq tracks from the Cistrome Data Browser database (http://cistrome.org/db/#/) showing the enrichments of H3K4me3, H3K27ac, and YY1 across the DICER1 promoter sequence. **C** Relative enrichment of YY1 (left panel) to DICER1 promoter was detected via ChIP assays using anti-YY1 antibody in BxPC-3 cells. The PCR products were run on a 2% agarose gel (right panel). **D** Schematic illustration showing the different mutants of YY1 binding sites of DICER1 promoter. **E** The luciferase reporter assays showing the relative activity of YY1 on DICER1 promoter in BxPC-3 cells using wild type (WT) or mutant-type (MUT)-containing promoter reporter vector. **F** Real-time qRT-PCR (upper panel) and western blot (upper panel) showing the changes of mRNA and protein levels of DICER1 in BxPC-3 and PANC-1 cells transfected with vector control, YY1, siNC, or siYY1 (#1, #2). **G** The luciferase reporter assays showing the relative activity of DICER1 promoter in BxPC-3 cells containing WT or MUT reporter vector. These cells were further transfected with vector, DICER1-AS1 and those co-transfected with siNC or siYY1. **H** Relative enrichment of YY1 to DICER1 promoter was detected via ChIP assays in PC cells transfected with empty vector, DICER1-AS1, and those co-transfected with siNC or siYY1. **I** Real-time qRT-PCR (left panel) and western blot (right panel) showing the changes of transcript and protein levels of DICER1 in BxPC-3 and PANC-1 cells transfected with vector or DICER1-AS1 plasmids and those co-transfected with siNC or siYY1. **J-K** The relative glucose uptake and relative lactic acid production were detected in PC cells transfected with empty vector or DICER1-AS1, and those co-transfected with siNC or siYY1. All data were presented as means ± SD of at least three independent experiments. Values are significant at ^a^P < 0.05, ^b^P < 0.01 and ^c^P < 0.001 as indicated

According to the analysis from the JASPAR database, the promoter area of the DICER1 gene possesses two potential binding positions for transcription factor YY1 (Fig. 5A). Importantly, the Cistrome Data Browser database [21] showed a significant enhancement of YY1 at the DICER1 promoter region accompanied by enriched peaks of histone modifications (H3K4me3 and H3K27ac) which indicating an active transcription (Fig. 5B). Furthermore, analysis of histone transcriptional markers of activation revealed that DICER1-AS1 overexpression increased, while DICER1-AS1 knockdown decreased the interaction of H3K4me3 and H3K27ac with DICER1 promoter (Additional file 3: Figure S7A-B). These results strongly suggested that DICER1-AS1 may have a potential role in regulating DICER1 transcription. Moreover, ChIP analysis confirmed that YY1 was accumulated in the P2 site of the DICER1 promoter (Fig. 5C). To verify the influence of YY1 on DICER1 transcription, we detected the luciferase activities of DICER1 promoter vectors containing the wild-type (WT) or mutant (MUT1-3) binding sites (Fig. 5D). The luciferase assays demonstrated that YY1 overexpression or knockdown could increase or decrease the luciferase intensity in PC cells transfected with WT plasmid and MUT1 plasmid, which further confirmed that the P2 site of the DICER1 promoter is necessary for YY1 to interact with DICER1 promoter (Fig. 5E). Coincidently, overexpression of YY1 increased, while silencing of YY1 decreased the expression of DICER1 (Fig. 5F). Furthermore, silencing of YY1 reversed the effects of DICER1-AS1 overexpression, while overexpression of YY1 rescued the effects of DICER1-AS1 knockdown on DICER1 promoter activity, YY1 accumulation on DICER1 promoter, DICER1 expression, as well as glucose uptake and lactate production in PC cells (Fig. 5G-K, Figure S7C-H). These data indicated that DICER1-AS1 promotes DICER1 transcription by recruiting YY1 to the DICER1 promoter.

### miR-5586-5p is an essential target for DICER1-AS1/DICER1 pathway

**Fig. 6:**
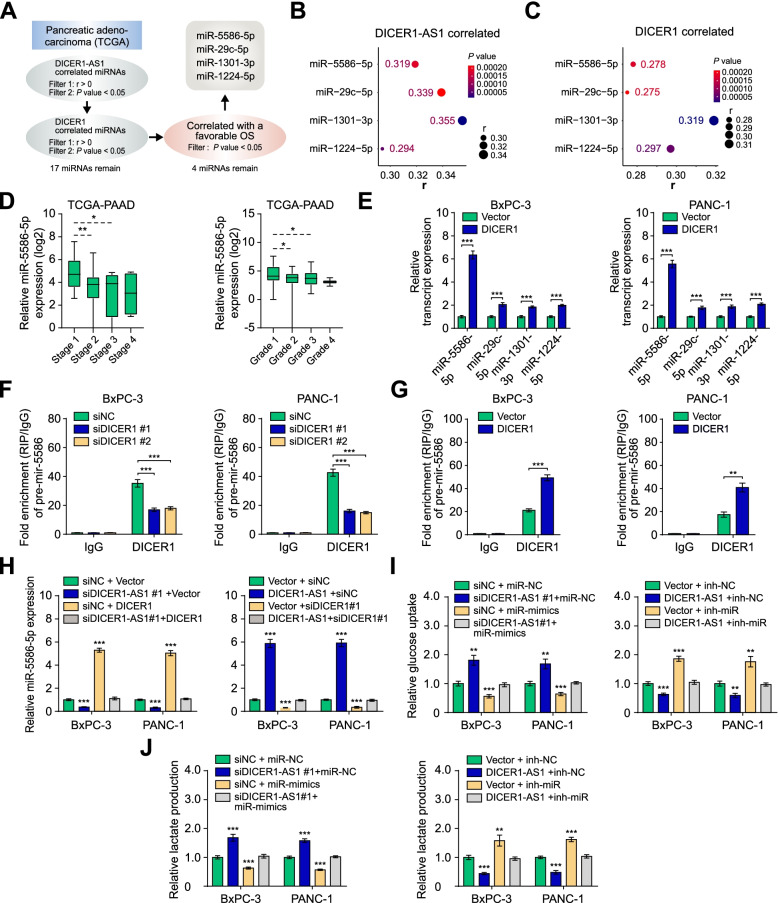
miR-5586-5p is an essential target for the DICER1-AS1/DICER1 pathway. A The flow chart for selected candidate miRNAs. B Bubble plots showing the expression correlation between DICER1-AS1 and candidate miRNAs. **C** Bubble plots showing the expression correlation between DICER1 and candidate miRNAs. **D** Boxplots showing the miR-5586-5p level in PC tissues with different statuses of stages and grades. **E** Real-time qRT-PCR showing the levels of candidate miRNAs in BxPC-3 and PANC-1 cells transfected with control vector or DICER1 plasmids. **F** RIP assays showing the binding efficiency between DICER1 protein and pre-mir-5586 using the anti-DICER1 antibody in BxPC-3 and PANC-1 cells transfected with siNC or siDICER1 (#1, #2). **G** RIP assays showing the binding efficiency between DICER1 protein and pre-mir-5586 using the anti-DICER1 antibody in BxPC-3 and PANC-1 cells transfected with vector control or DICER1 plasmids. **H** Real-time qRT-PCR showing the levels of miR-5586-5p in BxPC-3 and PANC-1 cells transfected with siNC, siDICER1-AS1, empty vector, or DICER1, and those co-transfected with empty vector, DICER1, siNC, or siDICER1. **I-J** The relative glucose uptake and the relative lactic acid production were detected in PC cells transfected with siNC, siDICER1-AS1, empty vector, or DICER1-AS1, and those co-transfected with miR-NC, miR-5586-5p mimics, inh-NC, or miR-5586-5p inhibitors. All data were presented as means ± SD of at least three independent experiments. Values are significant at ^a^P < 0.05, ^b^P < 0.01 and ^c^P < 0.001 as indicated

Since DICER1 plays an important role in the biogenesis of miRNAs, we presumed whether specific miRNAs might be contributing to the DICER1-AS1/DICER1 pathway. To determine the putative target miRNA, we analyzed the miRNAs positively correlated with expression of DICER1-AS1 and DICER1, as well as overall survival of PC in the TCGA database (Fig. 6A). The overlapping analyses indicated that miR-5586-5p, miR-29c-5p, miR-1301-3p, and miR-1224-5p were positively correlated with both DICER1-AS1 and DICER1 (Fig. 6B-C). Additionally, these 4 miRNAs were associated with favorable overall survival and relapse-free survival (Additional file 3: Figure S8A-B), and negatively associated with advanced clinical stages and grades (Fig. 6D, Additional file 3: Figure S8C-E). Although without obvious alteration of pre-miRNAs of the four miRNAs (Additional file 3: Figure S8F-G), knockdown or overexpression of DICER1 significantly decreased or increased expression of the 4 miRNAs in PC cells (Fig. 6E, Additional file 3: Figure S8H). Among those miRNAs, miR-5586-5p displayed the most significant change, then we selected miR-5586-5p as the critical target for further investigation. Consistently, RIP assays displayed that knockdown of DICER1 significantly decreased, but overexpression of DICER1 increased the binding of DICER1 to pre-mir-5586 (Fig. 6F-G). Moreover, overexpression or knockdown of DICER1 prevented the alteration of miR-5586-5p expression induced by the knockdown or overexpression of DICER1-AS1 (Fig. 6H). And miR-5586-5p was downregulated in PC tissues compared with normal tissues. (Additional file 3: Figure S8I). Meanwhile, miR-5586-5p mimics or inhibitors successfully reversed the changes of glucose uptake and lactate production in PC cells which were induced by knockdown or overexpression of DICER1-AS1 (Fig. 6I-J, Additional file 3: Figure S8J). Subsequently, analysis of TCGA database showed that the miR-5586-5p level was negatively associated with the tumor stage (Additional file 2: Table S5). Univariate and multivariate regression analyses showed that miR-5586-5p expression was an independent predictor of PC patients with significant hazard ratios for predicting favorably clinical outcomes (Additional file 2: Table S6). Collectively, these data demonstrated that miR-5586-5p is a critical target of DICER1-AS1/DICER1 pathway and functions as an inhibitor in pancreatic cancer.

### MiR-5586-5p regulates glycolysis by inducing mRNA degradation of glycolytic genes

**Fig. 7:**
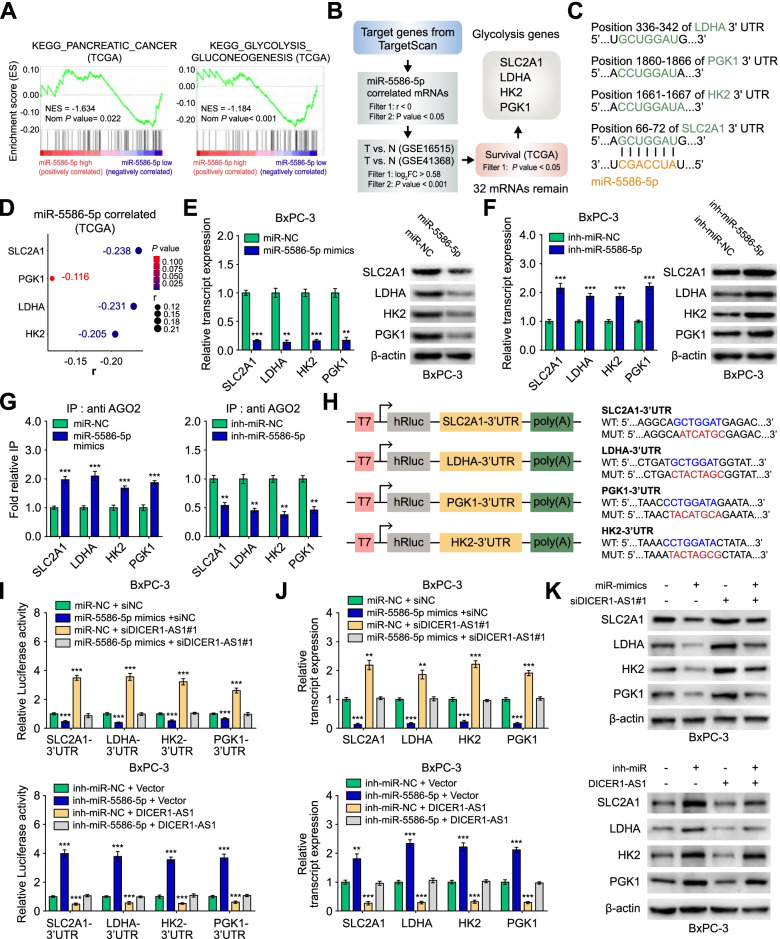
miR-5586-5p regulates glycolysis by inducing mRNA degradation of glycolytic genes. A GSEA analysis of miR-5586-5p-correlated genes derived from PC tissues (TCGA). NES, normalized enrichment score. **B** The flow chart for selected candidate glycolytic genes of miR-5586-5p target genes from the TargetScan database (http://www.targetscan.org/mamm_31/), and associated with overall survival of PC patients in TCGA database, and over-lapping analysis with genes differentially expressed (*P* < 0.001, Fold change > 1.5) in GEO database (GSE16515, GSE41368). **C** Schematic illustration showing the base pairing between miR-5586-5p and the 3’UTRs of glycolytic genes (SLC2A1, HK2, PGK1, LDHA) predicted by TargetScan database. **D** Bubble plots showing the expression correlation between miR-5586-5p and candidate glycolytic genes in PC tissues from the TCGA database. **E**–**F** Real-time qRT-PCR (left panel) and western blot (right panel) showing the levels of candidate glycolytic genes in BxPC-3 cells transfected with miR-NC, miR-5586-5p mimics, inh-miR-NC, or inh-miR-5586-5p. **G** RIP assays showing the effect of miR-5586-5p on the interaction of AGO2 with candidate glycolytic genes in PC cells using anti-AGO2 antibody. IgG was used as a negative control. **H** Schematic illustration showing the luciferase reporter constructs used for examining the effects of miR-5586-5p on the 3’UTR (WT, MUT) of SLC2A1, LDHA, HK2, and PGK1. WT: wild type; MUT: mutant type. **I** The dual-luciferase assay showing the luciferase reporter activity of 3’UTR of candidate glycolytic genes in BxPC-3 cells transfected with miR-NC, miR-5586-5p mimics, inh-miR-NC or inh-miR-NC, and those co-transfected with siNC, siDICER1-AS1, empty vector, or DICER1-AS1. **J-K** qRT-PCR and western blot showing the levels of glycolytic genes in BxPC-3 cells transfected with miR-NC, miR-5586-5p mimics, inh-miR-NC, or inh-miR-NC, and those co-transfected with siNC, siDICER1-AS1, empty vector, or DICER1-AS1. All data were presented as means ± SD of at least three independent experiments. Values are significant at ^a^P < 0.05, ^b^P < 0.01 and ^c^P < 0.001 as indicated

Since GSEA analysis revealed that miR-5586-5p was negatively correlated with the gene set of pancreatic cancer and glycolysis (Fig. 7A), we further investigated whether miR-5586-5p can regulate glycolytic gene expression. To test this possibility, we first performed the bioinformatics analysis by using the TargetScan database and then performed overlapping analyses with TCGA and GEO databases (GSE16515, GSE41368). The analysis demonstrated 32 potential targets of miR-5586-5p were overexpressed in PC tissues and correlated with poor survival (Fig. 7B), which including 4 glycolytic genes SLC2A1, LDHA, HK2, and PGK1 (Additional file 3: Figure S9A-C). The miR-5586-5p binding sites of the 4 glycolytic genes were predicted by the TargetScan database (Fig. 7C). As shown in Fig. 7D, four glycolytic genes (SLC2A1, LDHA, HK2, PGK1) were negatively correlated with miR-5586-5p.

Furthermore, miR-5586-5p mimics substantially reduced, whereas miR-5586-5p inhibitors increased the mRNA and protein levels of SLC2A1, LDHA, HK2, and PGK1 (Fig. 7E-F, Additional file 3: Figure S9D-E). Since AGO2 participates in the formation of the RISC complex, we further investigated whether miR-5586-5p affects the binding of AGO2 with target mRNAs. RIP assays indicated that mimics or inhibitors of miR-5586-5p significantly facilitated or repressed the interaction of AGO2 with SLC2A1, LDHA, HK2, and PGK1 (Fig. 7G). Meanwhile, we found that mimics or inhibitors of miR-5586-5p significantly increased or reduced the degradation of SLC2A1, SLC2A1, HK2, and PGK1 mRNAs in BxPC-3 cells treated with actinomycin D (Additional file 3: Figure S9F). To further verify these glycolytic genes are the direct target of miR-5586-5p, we constructed luciferase reporter plasmids containing wild type or mutant type 3’UTR (WT, MUT) of these genes (Fig. 7H). Luciferase assays indicated that miR-5586-5p mimics decreased, but the inhibitors increased the reporter activities, which was further reversed by knockdown or overexpression of DICER1-AS1 (Fig. 7I). Moreover, qRT-PCR and western blot assays confirmed that knockdown of DICER1-AS1 relieved the inhibition of miR-5586-5p mimics on the glycolytic genes, whereas overexpression of DICER1-AS1 deprived the upregulation of miR-5586-5p inhibitors on the glycolytic genes (Fig. 7J-K). Collectively, these results demonstrated that miR-5586-5p regulates the stability of the mRNAs of glycolytic genes to participate in the DICER1-AS1/DICER1 pathway.

### N6-methyladenosine reader YTHDF3 decreases DICER1-AS1 stability of PC cells in response to glucose deprivation

**Fig. 8:**
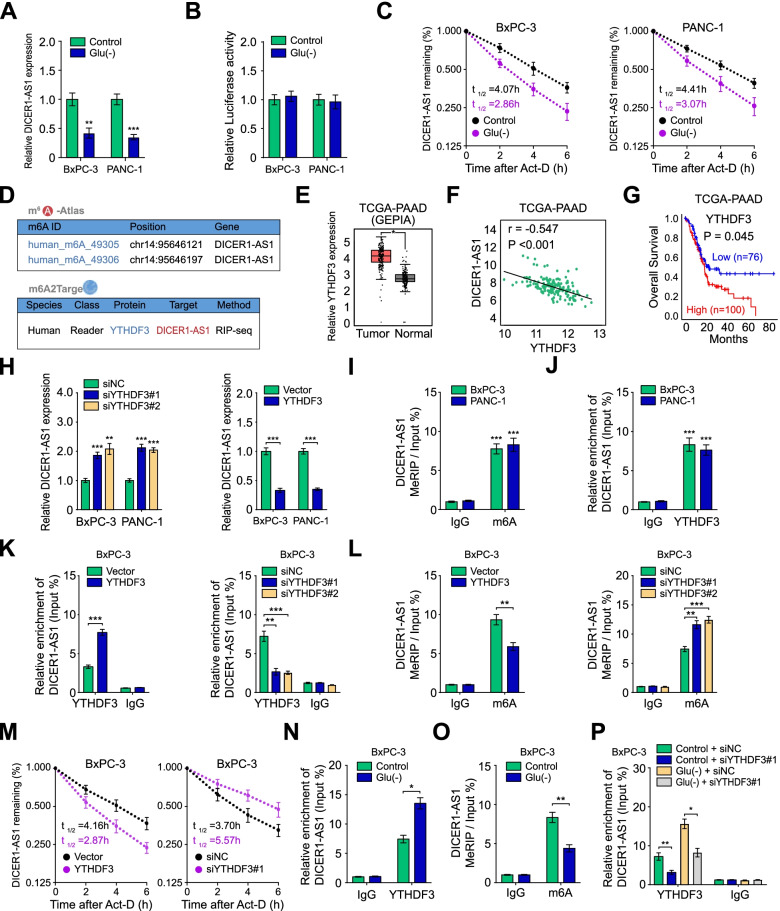
m6A reader YTHDF3 decreases DICER1-AS1 stability of PC cells in response to glucose deprivation. A The DICER1-AS1 level was detected by qRT-PCR in BxPC-3 and PANC-1 cells under glucose starvation (Glu(-)). **B** Luciferase activity of BxPC-3 and PANC-1 cells transfected with pGL3 reporter vector containing DICER1-AS1 promoter was measured during normal or glucose starvation environment. **C** The effects of glucose starvation on the RNA half-life (t1/2) time of DICER1-AS1 in PC cells. **D** Online bioinformatics tools m6A2Target database (http://m6a2target.canceromics.org/#/) and m6A-Atlas database (http://180.208.58.66/m6A-Atlas/index.html) suggested that DICER1-AS1 has m6A sites and was the potential target of YTHDF3. **E** Boxplot showing the differential expression of YTHDF3 between PC tissues and normal tissues from the GEPIA database (http://gepia.cancer-pku.cn/index.html). **F** The expression correlation between YTHDF3 and DICER1-AS1 of PC tissues from the TCGA database. **G** Kaplan–Meier curves showing the survival of PC patients from the TCGA database with a low and high level of YTHDF3 using a log-rank test (cutoff value = 2819.87). **H** After knockdown or overexpression of YTHDF3, the expression of DICER1-AS1 was evaluated by qRT-PCR in BxPC-3 and PANC-1 cells. **I** The MeRIP‐qPCR showed the m6A modification was highly enriched within DICER1-AS1 in PC cells. **J** RIP assay showed the enrichment of DICER1-AS1 with YTHDF3 protein by using the anti-YTHDF3 antibody in PC cells. And the co-precipitated RNA was subjected to qRT-PCR for DICER1-AS1. **K** RIP assay showed the effects of YTHDF3 on the enrichment of DICER1-AS1 with YTHDF3 protein. **L** The MeRIP‐qPCR showed the effects of YTHDF3 on the enrichment of m6A with DICER1-AS1. **M** The effects of YTHDF3 on the RNA half-life (t1/2) time of DICER1-AS1 in actinomycin D-treated PC cells. **N** RIP assay showed the enrichment of DICER1-AS1 on YTHDF3 protein in BxPC-3 cells cultured in a normal or glucose-free medium for 48 h. **O** The MeRIP‐qPCR showed the m6A modification level of DICER1-AS1 in BxPC-3 cells cultured in a normal or glucose-free medium for 48 h. **P** RIP assay showed the enrichment of DICER1-AS1 on YTHDF3 protein in BxPC-3 cells transfected with siNC or siYTHDF3#1 in the normal or glucose-free medium. All data were presented as means ± SD of at least three independent experiments. Values are significant at ^a^*P* < 0.05, ^b^*P* < 0.01 and ^c^*P* < 0.001 as indicated

Numerous lncRNAs were aberrantly expressed for adaption to nutrient stress in tumor microenvironment, including hypoxia, acidity, glutamine deprivation, and glucose deprivation [22, 23]. Interestingly, DICER1-AS1 was only notably decreased during glucose deprivation, but without significant alteration under hypoxia, acidity, and glutamine deprivation in PC cells (Fig. 8A, Additional file 3: Figure S10A). To further investigate whether the glucose deprivation induced downregulation of DICER1-AS1 of PC cells, the luciferase reporter assay was performed. Basic pGL3 plasmid and pGL3 plasmid containing DICER1-AS1 promoter was transfected into BxPC-3 and PANC-1 cells. The results revealed that the luciferase intensity was not changed in both PANC-1 and BxPC-3 cells, indicating that DICER1-AS1 might be regulated at the post-transcriptional level during glucose deprivation (Fig. 8B). Nevertheless, accelerated RNA decay of DICER1-AS1 upon glucose starvation was confirmed in PC cells (Fig. 8C).

Since research had referred that m6A modification contributes to degradation of lncRNAs [24], we then wonder whether the enhanced degradation of DICER1-AS1 during glucose deprivation was mediated by m6A modification. According to the online tool m6A-Atlas, a high-confidence collection of reliable m6A sites [25], DICER1-AS1 has m6A sites in the exon region (at Chr14: 95,646,121, 95,646,197). Meanwhile, bioinformatics database m6A2Target (http://m6a2target.canceromics.org/#/) also demonstrated that DICER1-AS1 interacted with a m6A reader YTH-domain family member 3 (YTHDF3) (Fig. 8D). Meanwhile, analysis of TCGA database showed that YTHDF3 was significantly increased in pancreatic cancer tissues, as well as negatively correlated with DICER1-AS1 expression and overall survival (Fig. 8E-G). Then we found that knockdown or overexpression of YTHDF3 increased or decreased the expression of DICER1-AS1 (Fig. 8H, Additional file 3: Figure S10B). Besides, overexpression or knockdown of YTHDF3 increased or decreased the mRNA and protein levels of glycolytic genes (SLC2A1, LDHA, HK2, PGK1). Meanwhile, the expression of DICER1 was reduced or increased by YTHDF3 overexpression and knockdown, respectively (Additional file 3: Figure S10C-D).

To validate the results of m6A databases, we detected the m6A modification of DICER1-AS1 in PC cells by the MeRIP-qPCR assay. The results showed that DICER1-AS1 was enriched with m6A antibody in both PANC-1 and BxPC-3 cells (Fig. 8I). RIP assay with YTHDF3 antibody further verified that YTHDF3 bonded to DICER1-AS1 to form a m6A modification complex (Fig. 8J). In addition, with increased interaction between YTHDF3 and DICER1-AS1 induced by YTHDF3 overexpression, YTHDF3 overexpression decreased m6A enrichment and stability of DICER1-AS1. On the contrary, the m6A enrichment and stability of DICER1-AS1 were enhanced by knockdown of YTHDF3, accompanied by decreased interaction between YTHDF3 and DICER1-AS1 (Fig. 8K-M, Additional file 3: Figure S10E-G). Coincidently, the RIP and MeRIP assay further revealed that glucose deprivation notably increased interaction between YTHDF3 and DICER1-AS1, which consequently decreased m6A enriched DICER1-AS1 (Fig. 8N-O, Additional file 3: Figure S10H-I). Furthermore, the glucose deprivation-enhanced interaction between DICER1-AS1 and YTHDF3 was significantly inhibited by YTHDF3 knockdown, while the decreased stability of DICER1-AS1 during glucose deprivation was obviously increased after YTHDF3 depletion (Fig. 8P, Additional file 3: Figure S10J-K). Coincidently, MeRIP assay showed the inhibition of the m6A enrichment of DICER1-AS1 under glucose deprivation was reversed by YTHDF3 knockdown (Additional file 3: Figure S10L). Taken together, our data revealed that YTHDF3 decreases DICER1-AS1 stability via a m6A dependent manner in response to energy stress.

### YHDF3 is a reciprocal target for DICER1-AS1 via miR-5586-5p-induced inhibition

Furthermore, we detected whether YTHDF3 expression was affected in response to glucose deprivation. Interestingly, both YTHDF3 mRNA and protein expression were increased during glucose deprivation (Additional file 3: Figure S11A). Meanwhile, we found that miR-5586-5p was downregulated in BxPC-3 and PANC-1 cells under glucose deprivation (Additional file 3: Figure S11B). Moreover, TargetScan predicted a binding site for miR-5586-5p in 3’UTR of YTHDF3, which suggested that YTHDF3 would be mediated by miR-5586-5p. And the reporter plasmid harboring the wild type (WT) or mutant type (MUT) 3’UTR region of YTHDF3 was constructed and transfected into BxPC-3 cells to perform luciferase reporter assay (Additional file 3: Figure S11C). The results showed that miR-5586-5p mimics or inhibitors significantly decreased or increased the luciferase reporter activity in WT cells, but without alteration in MUT cells (Additional file 3: Figure S11D-E). Moreover, miR-5586-5p mimics or inhibitors significantly reversed or enhanced the YTHDF3 expression under glucose deprivation (Additional file 3: Figure S11F-G). The luciferase reporter assay showed miR-mimics reversed the decrease of luciferase density of WT cells which was affected in response to glucose deprivation (Additional file 3: Figure S11H). Therefore, these results implicated that YTHDF3 is a critical target of miR-5586-5p.

To explore the role of YTHDF3 and miR-5586-5p on the DICER1-AS1 expression during glucose deprivation, PC cells were transfected with vector or YTHDF3, and those co-transfected with miR-5586-5p mimics or inhibitors. The results showed that YTHDF3-mediated inhibition of DICER1-AS1 level was neutralized by miR-5586-5p mimics (Additional file 3: Figure S11I). Furthermore, YTHDF3 knockdown increased the DICER1-AS1 level, which was reversed by miR-5586-5p inhibitors (Additional file 3: Figure S11J). Together, these data indicate a reciprocal feedback of YTHDF3 and DICER1A-S1/miR-5586-5p in PC cells in response to glucose deprivation.

### YTHDF3/DICER1-AS1/DICER1/miR-5586-5p axis is pivotal for glycolysis and tumorigenesis of PC

**Fig. 9:**
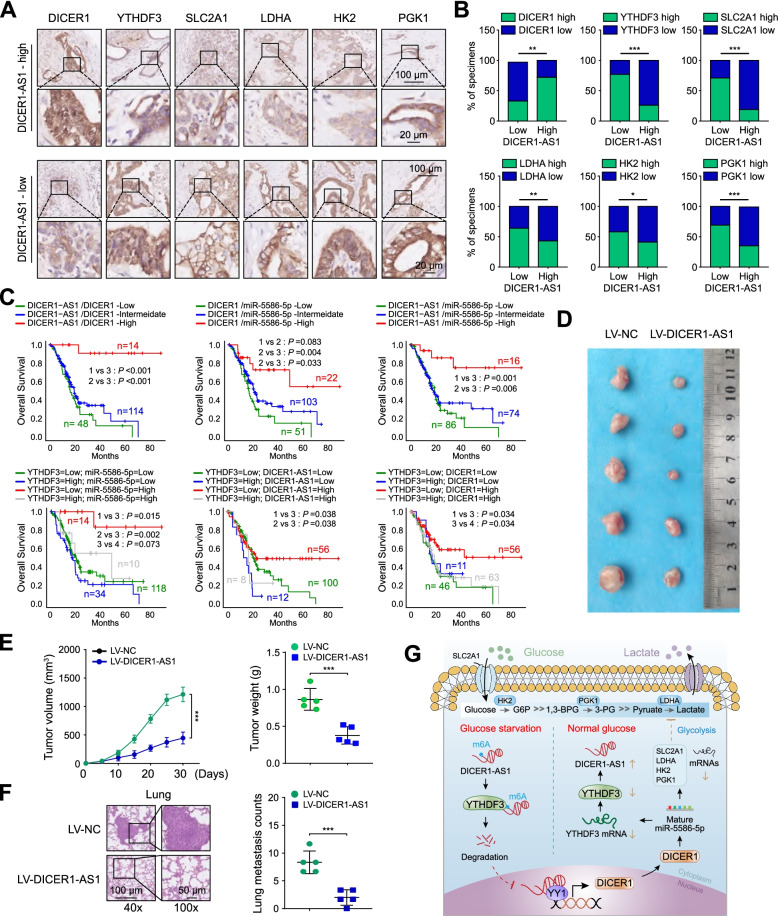
YTHDF3/DICER1-AS1/DICER1/miR-5586-5p axis is pivotal for glycolysis and tumorigenesis of PC. A The representative IHC images of DICER1, YTHDF3, SLC2A1, LDHA, HK2, and PGK1 in PC tissues (n = 86) with low or high levels of DICER1-AS1. Scale bar, 100 μm. **B** Statistical analyses (Chi-square test) showing the different levels of DICER1, YTHDF3, SLC2A1, LDHA, HK2, and PGK1 in PC tissues with high or low DICER1-AS1 levels. **C** Kaplan–Meier analysis (log-rank test) showing the OS curves based on the different groups of PC patients (TCGA database) with different levels of DICER1-AS1, DICER1, miR-5586-5p, and YTHDF3. **D** The images of xenografts formed by subcutaneous injection of BxPC-3 cells transfected with LV-NC or LV-DICER1-AS1. **E** The volume of the subcutaneous tumor was measured every 5 days in indicated groups (left panel). The weight of subcutaneous tumor was measured in the indicated groups after mice were sacrificed (right panel). **F** The representative HE staining images (left panel) and quantification (right panel) of lung tissues were isolated from indicated groups. The nude mice were treated with tail vein injection of BxPC-3 cells transfected with LV-NC or LV-DICER1-AS1. **G** Schematic representation for the mechanism of YTHDF3/DICER1-AS1/DICER1/miR-5586-5p axis as a switch that regulates glycolysis in pancreatic cancer by stabilizing the glycolytic genes under glucose deprivation. All data were presented as means ± SD of at least three independent experiments. Values are significant at ^a^P < 0.05, ^b^P < 0.01 and ^c^P < 0.001 as indicated

To further validate the function of DICER1-AS1 and its pathway in glycolysis of PC cells, we next performed immunohistochemical staining in 86 PC patients’ tissues of cohort 1. Consistently, the samples with low expression of DICER1-AS1 showed strong staining for YTHDF3 and glycolytic genes (SLC2A1, LDHA, HK2, PGK1), as well as weak staining of DCIER1. Whereas samples with high expression of DICER1-AS1 showed strong staining for DICER1, but weak staining for YTHDF3 and glycolytic genes (Fig. 9A-B). Kaplan–Meier survival analyses from TCGA database also indicated that low expression of DICER1-AS1/DICER1, DICER1/miR-5586-5p, and DICER1-AS1/miR-5586-5p groups showed a poor overall survival of PC patients. Meanwhile, low expression of YTHDF3 accompanied by high expression of miR-5586-5p, DICER1-AS1, or DICER1 showed a long overall survival of PC patients (Fig. 9C). To further evaluate the biological function of DICER1-AS1 in vivo, BxPC-3 cells were transfected stably by using lentivirus containing negative control (LV-NC) or DICER1-AS1 sequences (LV-DICER1-AS1). These transfected cells were further implanted into nude mice to construct a subcutaneous xenograft tumor model. The results revealed that the stable expression of DICER1-AS1 into BxPC-3 cells resulted in retarded growth and lower tumor weight of subcutaneous xenograft tumors (Fig. 9D-E). To evaluate the ﻿tumor metastasis in vivo, the nude mice were received tail vein injections ﻿with transfected BxPC-3 cells. We found that the number of lung metastases in the LV-DICER1-AS1 group was decreased compared to the control group (Fig. 9F). Correspondingly, the DICER1-AS1 transfected tumors displayed upregulation of DICER1-AS1, DICER1, and miR-5586-5p, as well as downregulation of YTHDF3, SLC2A1, LDHA, HK2, and PGK1 (Additional file 3: Figure S12A-D). Collectively, these results suggested that YTHDF3/DICER1-AS1/DICER1/miR-5586-5p axis is pivotal for glycolysis and tumorigenesis of pancreatic cancer.

## Discussion

Recently, the non-coding RNAs (ncRNAs), including miRNAs and lncRNAs have received growing attention in human tumors [26], particularly in pancreatic cancer [8, 27, 28]. Although studies have evaluated the involvement of lncRNAs in the dysregulation of glycolysis metabolism in pancreatic cancers, the precise mechanism needs to be further investigated [29, 30]. In this study, we identify that DICER1-AS1 is an inhibitor for glycolysis and tumorigenesis of pancreatic cancer by inducing miRNA-mediated mRNA degradation of glycolytic genes. Moreover, the present study indicates that DICER1-AS1 is epigenetically downregulated by its target m6A reader YTHDF3, which forms a negative feedback.

The bioinformatic analyses showed that DICER1-AS1 was downregulated in PC tissues and negatively correlated with glycolytic pathway and overall survival. We further identified that overexpression of DICER1-AS1 significantly inhibited glycolysis and consequently prohibited proliferation and metastasis of pancreatic cancer cells both in vitro and in vivo. Moreover, we further displayed that DICER1-AS1 was downregulated in PC tissues in our cohort 1 and associated with poor overall survival of PC patients. Thus, our study implicates that DICER1-AS1 is an inhibitor for glycolysis and tumorigenesis of pancreatic cancer. Differently, research from Gu et al. reported that DICER1-AS1 promoted the proliferation, invasion, and autophagy of osteosarcoma cells via miR-30b/ATG5 axis [31]. Other research also indicated that DICER1-AS1 functioned as promoter in colorectal cancer by regulating miR-296-5p/STAT3 pathway [32]. Therefore, these researches implicate that the function of DICER1-AS1 in heterogeneous cancer, which needs further investigation.

Natural antisense transcripts are a cluster of RNAs that are transcribed from the opposite strand of a sense DNA strand. It’s reported that antisense RNA can alter the expression of sense genes at the transcriptional or post-transcriptional level [33, 34]. Similarly, our present study demonstrated the inhibition on glycolysis of DICER1-AS1 was dependent on regulating DICER1 transcription. Meanwhile, as a critical processor for maturation of miRNA, we further founded that DICER1 promoted maturation of miR-5586-5p. Research also showed that knockdown of DICER1 significantly inhibited xenograft tumor growth of BxPC-3 cells by suppressing expression of miR-22, miR-143, let-7i, and miR-29b [35]. PICK1 inhibits the processing of pre-mir-615-3p to mature miR-615-3p via interfering with the binding of DICER1 to Smad2/3 in breast cancer cells [36]. Thereby, our research provides a new clue for interaction between lncRNA and miRNA which involving DICER1-mediated miRNA maturation.

YY1 is a ubiquitously distributed transcription factor belonging to the GLI-Krüppel family of zinc-finger protein, associated with multiple cellular processes [37]. YY1 suppressed the proliferation via inhibiting the transcription of lncRNA SOX2OT, thereby decreased the expression of its target gene SOX2 in PC [38]. Other studies also showed that YY1 suppressed the proliferation, invasion, and metastatic properties of PC cells by downregulating MMP10 [39], as well as FER and MMP2 [40]. Similarly, our present study validated that DICER1-AS1 promoted DCIER1 transcription by recruiting YY1 to promoter of DICER1. Meanwhile, YY1 was downregulated in PC tissues and correlated with DICER1 expression and poor prognosis of PC patients. Therefore, these data also identify that YY1 functions as an inhibitor in PC by interacting with DICER1-AS1/DICER1 pathway.

It’s reported that miRNAs play a vital role in metabolic homeostasis including glycolysis in tumors. For example, circMAT2B upregulated PKM2 by functioning as a “sponge” of miR-338-3p, meanwhile promotes the glycolysis level and hepatocellular carcinoma progression [41]. Besides, cell-secreted miR-122 is associated with metastasis in breast cancer patients by inhibiting glycolysis-related pyruvate kinase [42]. The present study demonstrated that miR-5586-5p induced degradation of glycolytic genes including SLC2A1, LDHA, HK2, and PGK1 by binding to 3’UTR of each mRNA, which consequently inhibited glycolysis of PC cells. Meanwhile, GEO database displayed that the 4 glycolytic genes were highly expressed in PC tissues and negatively correlated with miR-5586-5p and overall survival of PC patients. Similarly, research from Bai et al. showed that a seven-miRNA expression-based prognostic signature including miR-5586-5p was downregulated in PC which might stratify patients with PC into low- and high-risk groups [43]. Thereby, these data intensively reminder that miR-5586 is an inhibitor of PC and provides further evidence that miRNAs play critical roles in glycolysis of tumor.

Recent studies showed that m6A modification, as the most abundant RNA modification, was responsible for the dysregulation of lncRNAs [44]. Previous studies showed that the stability of lncRNA GAS5 was destroyed by YTHDF3 and consequently promoted progression of colorectal cancer [24]. Similarly, our study showed glucose deprivation significantly induced degradation of DICER1-AS1, which was mediated by m6A reader YTHDF3. Interestingly, our research also displayed that YTHDF3 was a critical target for miR-5586-5p. To explore the comprehensive effects of YTHDF3/DICER1-AS1/DICER1/miR-5586-5p axis on the glycolysis and prognosis of PC, we performed IHC assay and Kaplan–Meier survival analyses. The results demonstrated that high expression of DICER1-AS1 correlated with high expression of DCIER1, but correlated with low expression of YTHDF3 and the four glycolytic genes. Moreover, high expression of DICER1-AS1 along with high expression of DICER1 or miR-5586 was associated with longer overall survival. On the contrary, high expression of YTHDF3 along with low expression of DICER1 or DICER1-AS1 demonstrated shorter overall survival. Moreover, the in vivo experiments further identified that overexpression of DICER1-AS1 significantly inhibited the growth and metastasis of PC tumors.

## Conclusion

In summary, these results revealed an m6A-modified DICER1-AS1 is involved in glycolysis of PC by regulating glycolytic genes expression through processing maturation of miR-5586-5p and implicates that DICER1-AS1 might be a favorable biomarker and therapeutic target for treatment of PC.

## Supplementary Information

**Additional file 1.** Supplementary methods.

**Additional file 2.** **Table S1.** The sequences of PCR primers. **Table S2.** The sequences for gene knockdown. **Table S3.** Clinical information of 86 cases PAAD patients (cohort 1). **Table S4.** Univariate and multivariate analyses of DICER1-AS1 level and overall survival (TCGA). **Table S5.** Correlation between miR-5586-5p expression and clinicopathological features in PAAD patients (TCGA). **Table S6.** Univariate and multivariate analyses of the miR-5586-5p level and overall survival (TCGA).

**Additional file 3. Figure S1.** DICER1-AS1 is downregulated in pancreaticcancer tissues and negatively correlated with glycolysis pathway. **Figure S2.** Characteristics of lncRNA DICER1-AS1. **Figure S3.**Knockdown of DICER1-AS1 promotes glycolysis, proliferationand metastasis of PC cells**. Figure S4. **The expression correlation between DICER1-AS1and DICER1 and Kaplan-Meier survival curves of DICER1 in tumor datasets. **Figure S5. **DICER1 is a critical targetfor DICER1-AS1 regulating glycolysis**. Figure S6. **The expressioncorrelation between YY1 and DICER1 in the CCLE databaseand Kaplan-Meier survival curves of YY1 in tumordatasets. **Figure S7. **DICER1-AS1 regulates DICER1 transcription byrecruiting YY1 to the promoter of DICER1. **Figure S8.**miR-5586-5pis an essential target for the DICER1-AS1/DICER1 pathway. **Figure S9. **miR-5586-5p regulates glycolysis by inducingmRNA degradation of glycolytic genes.

## Data Availability

All data generated or analyzed during this study are included either in this article or in the supplementary Materials and Methods, Tables, and Figures.

## Abbreviations

PC: Pancreatic cancer
NP: Noncancerous peritumoral tissues
lncRNAs: Long non-coding RNAs
RIP: RNA immunoprecipitation
FISH: Fluorescence in situ hybridization
MeRIP: Methylated RNA immunoprecipitation
ChIP: Chromatin immunoprecipitation
qRT-PCR: Quantitative real-time PCR
IHC: Immunohistochemistry
ATCC: American Type Culture Collection
NCCN: National Comprehensive Cancer Network
Co-IP: Co-immunoprecipitation
HPDE: Human pancreatic duct epithelial
TCGA: The Cancer Genome Atlas
GO: Gene ontology
KEGG: Kyoto Encyclopedia of Genes and Genomes
GSEA: Gene set enrichment analysis
CPAT: Coding-Potential Assessment Tool
CPC: Coding Potential Calculator
CCLE: Cancer Cell Line Encyclopedia
OS: Overall survival
DFI: Disease-free interval
PFI: Progression-free interval
DSS: Disease-specific survival
ROC: Receiver operating characteristic curve
PAAD: Pancreatic adenocarcinoma
m6A: N6-methyladenosine
Act-D: Actinomycin D
