# Supplementary material for: A reciprocal feedback between N6-methyladenosine reader YTHDF3 and lncRNA DICER1-AS1 promotes glycolysis of pancreatic cancer through inhibiting maturation of miR-5586-5p
Source: J Exp Clin Cancer Res. 2022 Feb 19;41:69. doi: 10.1186/s13046-022-02285-6 (PMC8857805; doi:10.1186/s13046-022-02285-6)
Supplement: Supplementary file 1 — Additional file 1. Supplementary methods. [file 13046_2022_2285_MOESM1_ESM.docx]

**Supplementary methods**

**Cell culture**

The cell lines (SW1990, AsPC-1, PANC-1, BxPC-3) of human pancreatic cancer were obtained from the American Type Culture Collection (ATCC, USA). The normal pancreatic ductal epithelial cell line (HPDE) was obtained from Bena Culture Collection (BNCC, China). All cell lines were grown at 37°C with 5% CO2 and were maintained in Roswell Park Memorial Institute (RPMI) 1640 (Gibco, USA) with 10% fetal bovine serum (HyClone, USA). Anaerobic gas-producing bag (Hopebio, China) was used to build a hypoxia environment (1% oxygen). Cells were cultured under pH 6.8 for an acidic environment (Acidity). To build nutrient-deprived conditions, we incubated cells with a complete medium without glutamine (Glutamine (-)) or with 1mM glucose (Glucose (-)) for 48h, respectively. RPMI-1640 which without glutamine or glucose was purchased from Gibco, USA.

**RNA isolation, reverse transcription, and quantitative real-time PCR**

Total RNA was extracted from cells and tissues by using TRIzol reagent (TAKARA, Dalian, China). The reverse transcription of total RNA was performed by the PrimeScript® RT Master Mix Perfect Real-Time (TAKARA, Dalian, China). Quantitative real-time PCR was performed with the SYBR Premix Ex Taq II (TAKARA) by StepOnePlus real-time PCR system (Applied Biosystems, Foster City, CA). β-actin was used as an internal control gene, and the quantitative analysis of the relative expression levels was performed using the 2^-△△Ct^ method. All primers (Sangon, Shanghai) used were listed in Additional file 2: Table S1.

**Transfection**

The small interfering RNAs (siRNAs, 50 nM) against human DICER1-AS1, DICER1, YY1, and YTHDF3 were obtained from Ribobio (Guangzhou, China). The plasmids containing DICER1-AS1, DICER1, YY1, YTHDF3, control vector, and corresponding truncates were purchased from GeneChem (Shanghai, China). Cells were transfected with siRNAs using the transfection reagent of Lipofectamine 2000 (Invitrogen) and Opti-MEM I (Invitrogen) while scramble siRNA was used as a negative control. The miR-5586-5p mimic and negative control (NC) oligonucleotides, miR-5586-5p inhibitor, and scramble oligonucleotides were obtained from Ribobio (Guangzhou, China). The plasmids were transfected into cells using the transfection reagent of Lipofectamine 2000 (Invitrogen) and Opti-MEM I (Invitrogen). The lentiviral vectors containing a negative control sequence (LV-NC) or DICER1-AS1 sequence (LV-DICER1-AS1) were purchased from GeneChem (Shanghai, China). The sequences of siRNAs and vectors were shown in Additional file 2: Table S2.

**Patients and** **clinical samples**

The clinical samples including tumor tissues and matched noncancerous tissues were obtained from patients with pancreatic cancer who underwent surgical resection or biopsy of the palliative surgery including I^125^ seeds implantation as well as choledochojejunostomy and gastroenterostomy at the Pancreatic Disease Institute of Union Hospital (Wuhan, China). The fresh specimens were immediately frozen with liquid nitrogen after surgical resection and then stored at -80℃. The study was approved by the local Research Ethics Committee at the Academic Medical Center of Huazhong University of Science and Technology. And all procedures performed on patients according to the National Comprehensive Cancer Network (NCCN 2012) guideline for pancreatic cancer. All procedures of the study were performed following the Declaration of Helsinki. Meanwhile, written informed consent was obtained from all patients in this study. The clinical information of Cohort1 was listed in Additional file 2: Table S3.

**RNA** **fluorescence in situ hybridization (RNA-FISH) assay**

FISH Tag™ RNA Multicolor Kit (Invitrogen, USA) was used to detect lncRNA DICER1-AS1. The synthesis, labeling, and purification procedures of probes were according to the manufacturer’s instructions. In brief, cells or tissues were fixed in formaldehyde, permeabilized by Triton X-100, and then hybridization was performed using labeled probes in a moist chamber at 42°C overnight in the darkroom. Cells and tissues were added with a 2 × 10 μl antifade reagent containing 4,6-diamidino-2-phenylindole (DAPI) under coverslips. The fluorescence images were observed using the LSM 5 Pascal Laser Scanning Microscope (Zeiss, Germany). If necessary, the protein immunofluorescence assays were performed after the FISH assays were completed.

**MTT assay**

3 × 10^3^ cells were seeded in 96-well plates and observed for 5 days. After being cultured with 20 μl MTT (5 mg/ml) per well for 4 h in an incubator, the mix of MTT and medium was replaced by 150 μl DMSO (Sigma) each well. We observed the absorbance by ELISA reader (Thermo Fisher Scientific) around 570 nm.

**Wound healing assay**

Cells were plated in 12-well plates till full confluence. Then cells were scratched smoothly using a 10 µl pipette tip. After 48h, cells were washed carefully with PBS to remove cell debris. Then, images were taken by a light microscope.

**Transwell assay**

For invasion assays, cells resuspended with the serum-free medium were seeded in the top chambers (Corning Costar) coated with Matrigel (Sigma). Meanwhile, the bottom chambers were filled with 30% FBS as chemotaxis. After 48 h, the cells that failed to invade the membranes of the chambers were erased carefully. Then the invaded cells on the bottom of the membrane were fixed and stained with 0.05% crystal violet. Finally, 5 random fields (×40 magnifications) of the invaded cells were counted.

**Western blot assay**

Western blot assays were performed according to the protocol. The cell lysate containing 20-30 µg proteins was denatured and subjected to SDS-PAGE gel electrophoresis. Then proteins were transferred to PVDF membranes (Millipore) and probed with the following primary antibodies: β-actin (Proteintech, 20536-1-AP), DICER1 (Proteintech, 20567-1-AP), YY1 (Proteintech, 22156-1-AP), HuR (Proteintech, 11910-1-AP), SLC2A1 (Proteintech, 21829-1-AP), LDHA (Proteintech, 19987-1-AP), HK2 (Proteintech, 22029-1-AP), PGK1 (Proteintech, 17811-1-AP), YTHDF3 (Proteintech, 25537-1-AP).

**Xenograft assay**

For the xenograft assay, cells (2×10^6^ cells/mouse) suspended in 100 μl RPMI 1640 medium were subcutaneously implanted in the flank of nude BALB/c mice (4 weeks old; HFK Bio-Technology Co., Ltd, Beijing, China). There was a total of five mice per group and the tumor volume and mice weight were measured every 5 days. Tumor weight was measured at the end of the experiment. Mice were sacrificed at 28 days after cells were implanted. ﻿To investigate tumor metastasis, mice were received tail vein injections with 1×10^4^ transfected BxPC-3 cells. Then the ﻿tumor colony in lung was confirmed and counted after HE staining after 4 weeks. Our animal experiments were approved and reviewed by the Animal Research Committee of the Academic Medical Center at Huazhong University of Science and Technology. The care and handling of the animals were following the guidelines for Institutional and Animal Care and Use Committees.

**Glucose uptake assay**

Cells were plated into 24-well (5x10^4^ cells/well) plates and transfected with indicated constructs or siRNA and incubated for 24 hours. To measure glucose uptake, the transfected cells were treated with fluorescence-based 2-NBDG (APExBIO, USA) and 100 nM insulin for 30 min at 37°C according to the manufacturer’s instructions. The fluorescence intensity of 2-NBDG was measured by flow cytometry and normalized to total cellular protein concentration.

**Lactate Production Assay**

Cells were plated into 24-well (5x10^4^ cells/well) plates and transfected with indicated constructs or siRNA and incubated for 24 hours. To measure the production of lactate, the Lactic Acid assay kit (Jiancheng, China) was used according to the manufacturer’s instructions. The lactate production levels were measured at 530 nm by ELISA reader (Thermo Fisher Scientific) and normalized by the number of cells.

**Luciferase reporter assay**

DICER1 and DICER1-AS1 promoter region (2kb sequence upstream of the transcription initiation site) and corresponding mutations were inserted into pGL3-based vectors. 24 hours after transfection, firefly and renilla luciferase activity were measured by the dual-luciferase reporter system (Promega, USA). The reporter activity was normalized by the Renilla luciferase. To evaluate the effect of miR-5586-5p on mRNA 3’UTR of glycolysis genes (SLC2A1, LDHA, HK2, PGK1), pancreatic cancer cells were co-transfected with the psicheck2-based vectors containing mRNA 3’UTR of glycolysis genes and miR-5586-5p mimics or inhibitors, respectively. 24 hours after transfection, firefly, and renilla luciferase activity were measured by the dual-luciferase reporter system (Promega, USA). The reporter activity was normalized by the Renilla luciferase. The assays were performed three independent times.

**RNA stability assay**

For RNA stability assay, cells after transfection were treated with actinomycin D at a final concentration of 5μg/mL for 0, 3, and 6 h. Total RNAs were extracted and analyzed by PCR. The mRNA expression for each group at the indicated time was calculated using the 2^-△△Ct^ method and normalized by β-Actin. The mRNA half-live time was estimated according to the previous report. Briefly, the constant of mRNA decay (Kdecay), mRNA concentration at 0 hours (C_0_), transcription inhibition time (t), and mRNA concentration at t hour (C) leads to the following equation:

ln(C/C_0_) = -K_decay_t

Thus, the mRNA half-time (C_0_/C=0.5) can be estimated by the equation:

t_1/2_=In2/K_decay_

**Chromatin immunoprecipitation (ChIP)**

For the ChIP assay, we used the EZ-ChIPTM Chromatin Immunoprecipitation Kit (Millipore, Billerica, MA, USA) according to the manufacturer's instructions. In brief, cells were cross-linked with 1% formaldehyde for 10 min. To obtain the DNA fragments, cellular DNA was sonicated to an average size of 300-500 bp by ultrasonic instrument. Then, the lysates were mixed with the corresponding antibody to form the protein-DNA complex. After the procedure of purifying, the eluted DNA fragments were further detected via qRT-PCR using the specific primers. The Anti-IgG antibody was used as a negative control.

**RNA pull-down assay**

For RNA pull-down assay, RNA was synthesized in vitro via RT-PCR using specific primers containing a T7 RNA-polymerase promoter sequence according to the manufacturer's instructions of MAXIscript® Kit (Ambion, USA). Then the RNA was end-labeled with desthiobiotin by using Pierce RNA 3′ End Desthiobiotinylation Kit (Thermo Scientific, USA). Finally, RNA pull-down assay was performed using the Pierce™ Magnetic RNA-Protein Pull-Down Kit (Thermo Scientific, USA) according to the manufacturer's instructions. Briefly, the biotinylated DICER1-AS1 and its truncates or antisense-DICER1-AS1 (50 pmol) were incubated with protein lysate (2 mg) and then mixed with streptavidin beads (50 μl). After incubation and 3 times of wash, the beads were boiled in SDS buffer for following western blotting. The extracted total protein was used as a positive control.

**RNA-binding protein immunoprecipitation (RIP)**

RIP assays were conducted using the RNA-Binding Protein Immunoprecipitation Kit (Magna RIP™, Millipore, USA) according to the manufacturer’s instructions. Briefly, the lysate was incubated with RIP buffer containing magnetic beads coated with a specific antibody of interest (5 μg) overnight at 4 °C. And the normal rabbit IgG (5 μg) was used as a negative control. For the purify of RNA, the RNA-protein complexes were washed 3 times and incubated with 150ul proteinase K buffer. And 400u Phenol/Chloroform/Isoamyl-alcohol buffer was used for the RNA extraction. Finally, the precipitated RNA was detected by RT-PCR with specific primers

**MeRIP**

m6A modifications of RNAs were measured by methylated RNA immunoprecipitation (MeRIP) assay. Briefly, we purified poly(A)+ RNA by using VAHTSTM mRNA Capture Beads (Vazyme Biotech Co., China). RNAs were denatured to 70°C for 10 minutes followed by ice treatment. Then RNAs were incubated with m6A antibody (Abcam, UK) in 1ml buffer containing RNase inhibitor (Beyotime Institute of Biotechnology, China), 50 mM Tris-HCl, 750 mM NaCl, and 0.5% (vol/vol) Igepal CA-630 for 2 hr at 4°C. Protein G (Bimake, China) was washed, added to the mixture, and incubated for 2 hours at 4°C with rotation. m6A RNA was eluted twice with 6.7 mM N6-methyladenosine 5′-monophosphate sodium salt at 4°C for 1 hour and precipitated with 5 μg glycogen, one-tenth volume of 3 M sodium acetate in 2.5 volumes of 100% ethanol at −80°C overnight. m6A enrichment levels were measured by RT-PCR.

**Immunohistochemistry (IHC)**

For immunohistochemical staining, formalin-fixed tissues were embedded with paraffin, then sectioned (4 µm) for the following procedures. In brief, the tissues were mixed with EDTA Antigen Retrieval solution for antigen retrieval. The slides were incubated with specific antibodies in a darkroom at 4℃ overnight. DAB was used for the visualization of the immune complexes. Then hematoxylin was used for nucleus counterstaining. Finally, the protein expressions were assessed via the intensity and extent of staining at 200x under a light microscope.

**Immunoﬂourescence**

For immunoﬂuorescent staining, cells were seeded on overslips with 4% paraformaldehyde for 20 min, permeabilized with 0.5% TritonX-100 (10 min), and blocked with 5% Bovine Serum Albumin (Servicebio, China) for 40 min. And then, the cells were incubated with specific primary antibodies (1:300 dilution) at 4 °C overnight. FITC-conjugated goat anti-rabbit IgG (Jackson Immuno Research, USA) was used as a secondary antibody. And DAPI (Sigma, USA) was used to stain nuclei. The images were taken via Zeiss LSM510 microscopy (Germany).

**Gene set enrichment analysis (GSEA)**

To illustrate the function of genes concerning other genes, gene set enrichment analysis was performed using the GSEA software. The published gene sets were obtained from the official website (<https://www.gsea-msigdb.org/gsea/index.jsp>). To analyze the particular pathways or biologic processes of genes interested, the outcomes were generated from published data including the GEO microarray and TCGA database.

**Statistical analysis**

All statistical analyses were performed using R-3.0.2 software (<http://cran.r-project.org/bin/windows/base/old/3.0.2/>). All data were presented at least three independent experiments and were shown as means ± standard deviation (SD). The student’s *t****-***test or Mann-Whitney U test was used to compare the difference between the two groups when appropriate. And paired samples were analyzed using the paired t-test. χ2 analysis or Fisher exact probability analysis was used to analyze the clinical characteristics. Multiple comparisons were performed using the one-way ANOVA test. Spearman’s correlation coefficient was used to analyze the correlation between two genes. Kaplan-Meier analysis and a log-rank test were used to compare the different survival rates. Cox regression models were performed to assess survival differences and hazard ratios (HR). All statistical tests were two-sided.
