# Supplementary material for: A reciprocal feedback between N6-methyladenosine reader YTHDF3 and lncRNA DICER1-AS1 promotes glycolysis of pancreatic cancer through inhibiting maturation of miR-5586-5p
Source: J Exp Clin Cancer Res. 2022 Feb 19;41:69. doi: 10.1186/s13046-022-02285-6 (PMC8857805; doi:10.1186/s13046-022-02285-6)
Supplement: Supplementary file 2 — Additional file 2. Table S1. The sequences of PCR primers. Table S2. The sequences for gene knockdown. Table S3. Clinical information of 86 cases PAAD patients (cohort 1). Table S4. Univariate and multivariate analyses of DICER1-AS1 level and overall survival (TCGA). Table S5. Correlation between miR-5586-5p expression and clinicopathological features in PAAD patients (TCGA). Table S6. Univariate and multivariate analyses of the miR-5586-5p level and overall survival (TCGA). [file 13046_2022_2285_MOESM2_ESM.docx]

**Table S1. The sequences of PCR primers.**

| **Primer** | **Sequence** |
| --- | --- |
| DICER1-AS1 | Forward: 5′- ACAAGTTGGAGCTGAAGGAGTG -3′ |
|  | Reverse: 5′- AACCCTCACAACACATGGGC -3′ |
| DICER1  Pre-DICER1-AS1    miR-5586-5p  pre-miR-5586-5p  U6 snRNA  β-actin  pre-miR-29c  miR-29c  pre-miR-1301  miR-1301-3p  pre-miR-1224  miR-1224-5p  SLC2A1  LDHA  HK2  PGK1  GAPDH  YY1  YTHDF3  DICER1-CHIP1  DICER1-CHIP2  DICER1-AS1-  transcription in vitro  DICER1-AS1 probe | Forward: 5′- TGCAACTTGGTGGTTCGTTT -3′  Reverse: 5′- GCCGTGTTGATTGTGACTCG -3′  Forward: 5′- ACGAAGAAATGGAATA-3′  Reverse: 5′- AGCTACAAAGAAAACC-3′  TATCCAGCTTGTTACTATATGC  GCTTGTTACTATATGCTTTTTAAAT  Forward: 5′- CTCGCTTCGGCAGCACA-3′  Reverse: 5′- AACGCTTCACGAATTTGCGT-3  Forward: 5’-TAGCCGCCAAAGGTCCAATG-3’  Reverse: 5’-GATAAGCCCTACGAGCGACC-3’  TGGTGTTCAGAGTCTGTTTTTGTCTAGCACCATTTGAAA  ACCGATTTCTCCTGGTGTT  AGCACTGTGCTGGGGATGTTGC  AGCTGCCTGGGAGTGACTT  GGGAGGTGGAGGGTGGT  GTGAGGACTCGGGAGGT  Forward: 5′- ATTGGCTCCGGTATCGTCAAC-3′  Reverse: 5′- GCTCAGATAGGACATCCAGGGTA -3′  Forward: 5′- AGGCTATTCTTGGGCAACCC-3′  Reverse: 5′- TGAGTAGACATCCACCAAGGTT-3′  Forward: 5′-GTGAATCGGAGAGGTCCCAC-3′  Reverse: 5′-CAAGCAGATGCGAGGCAATC-3′  Forward: 5′-CCACTGTGGCTTCTGGCATA -3′  Reverse: 5′-ATGAGAGCTTTGGTTCCCCG-3′  Forward: 5′- GGAGCGAGATCCCTCCAAAAT-3′  Reverse: 5′- GGCTGTTGTCATACTTCTCATGG-3′  Forward: 5′- AACAGGCATCCCGAGTTCAG -3′  Reverse: 5′- GGGGGCTAAAATCACAGCCT-3′  Forward: 5′-TCAGAGTAACAGCTATCCACCA-3′  Reverse: 5′- GGTTGTCAGATATGGCATAGGCT-3′  Forward: 5′-GAGGCAGGTCAGGAGTTC-3′  Reverse: 5′- CTTGTTCCATCAGCAGGT-3′  Forward: 5′- GCTGTGCTGTTCAGTTTGGG-3′  Reverse: 5′- GCCAGACTACGTGGGTTCAA-3′  Forward: 5′- TAATACGACTCACTATAGGGACTGGACCTTGGCG -3′  Reverse: 5′- GCACAGCAGCTCCCCTGTCCTTCTCG -3′  Forward: 5′- TAATACGACTCACTATAGGGCTTAAGAGAGACAG-3′  Reverse: 5′- CAGAAGAGACGGGGTTTCACCATG-3′ |

**Table S2. The sequences for gene knockdown.**

| **SiRNA Targets** | **Sequences** |
| --- | --- |
| DICER1-AS1-siRNA#1 sense | 5′-GACGAAGAAATGGAATAACTTCC-3′ |
| DICER1-AS1-siRNA#2 sense | 5′-ATGGAATAACTTCCAACAAGTTG-3′ |
| DICER1-AS1-siRNA#3 sense | 5′-TGGAATAACTTCCAACAAGTTGG-3′ |
| DICER1-siRNA#1 sense | 5′- AAGAAGCAATTCATGATAACATT-3′ |
| DICER1-siRNA#2 sense | 5′- AGCACTTAATTTTATCAATGATT-3′ |
| DICER1-siRNA#3 sense  YY1-siRNA#1 sense  YY1-siRNA#2 sense  YTHDF3-siRNA#1 sense  YTHDF3-siRNA#2 sense | 5′-TTGCTATGTCGCCTTGAATGTTT-3′  5′-CTCAGATGAAAAAAAAGATATTG-3′  5′-GTGGTTGAAGAACAGATCATTGG-3′  5′-GACCTAAAGGGCAAGGAAATAAA-3′  5′-TCCATCCATTGGATTTCCATATT-3′ |
| NC-siRNA sense | 5′-TTCTCCGAACGTGTCACGTTT-3′ |

**Table S3. Clinical information of 86 cases PAAD patients (cohort 1).**

| id | Survival time(days) | Survival state | gender | age | size | stage | grade | DICER1-AS1 |
| --- | --- | --- | --- | --- | --- | --- | --- | --- |
| 49 | 4 | 1 | MALE | 67 | 2.46 | Ⅳ | 1 | -14.21 |
| 33 | 45 | 1 | FEMALE | 61 | 3.4 | Ⅰ | 4 | -12.52 |
| 14 | 23 | 0 | FEMALE | 45 | 1 | Ⅲ | 3 | -12.14 |
| 34 | 26 | 0 | FEMALE | 73 | 4.2 | Ⅳ | 2 | -11.43 |
| 53 | 163 | 1 | MALE | 68 | 2.3 | Ⅰ | 1 | -11.1 |
| 6 | 268 | 1 | FEMALE | 53 | 1.2 | Ⅰ | 1 | -10.84 |
| 86 | 1357 | 1 | FEMALE | 71 | 4.1 | Ⅲ | 1 | -10.6 |
| 60 | 900 | 0 | MALE | 62 | 3.2 | Ⅲ | 3 | -10.38 |
| 83 | 148 | 1 | MALE | 48 | 4 | Ⅱ | 1 | -10.21 |
| 1 | 88 | 0 | MALE | 65 | 3.2 | Ⅲ | 4 | -10.05 |
| 10 | 19 | 1 | FEMALE | 61 | 1.4 | Ⅲ | 3 | -9.98 |
| 67 | 556 | 1 | MALE | 58 | 4 | Ⅰ | 4 | -9.95 |
| 31 | 855 | 0 | FEMALE | 70 | 3 | Ⅳ | 1 | -9.9 |
| 61 | 149 | 1 | MALE | 74 | 3.1 | Ⅰ | 1 | -9.9 |
| 13 | 372 | 1 | FEMALE | 54 | 1.4 | Ⅱ | 3 | -9.5 |
| 62 | 981 | 0 | FEMALE | 73 | 4.1 | Ⅳ | 4 | -9.37 |
| 40 | 683 | 1 | MALE | 56 | 2.2 | Ⅲ | 2 | -9.28 |
| 42 | 571 | 0 | MALE | 44 | 2.3 | Ⅳ | 3 | -9.07 |
| 3 | 15 | 0 | MALE | 66 | 0.6 | Ⅲ | 2 | -8.94 |
| 43 | 487 | 1 | MALE | 57 | 3.1 | Ⅰ | 1 | -8.93 |
| 70 | 357 | 0 | FEMALE | 81 | 2.6 | Ⅲ | 2 | -8.91 |
| 8 | 1375 | 0 | FEMALE | 67 | 4.4 | Ⅳ | 4 | -8.88 |
| 11 | 821 | 1 | MALE | 54 | 3.7 | Ⅲ | 1 | -8.78 |
| 76 | 1149 | 1 | MALE | 67 | 1.9 | Ⅲ | 3 | -8.77 |
| 73 | 112 | 0 | FEMALE | 58 | 4.3 | Ⅲ | 4 | -8.73 |
| 22 | 396 | 1 | MALE | 77 | 3.3 | Ⅳ | 3 | -8.72 |
| 72 | 459 | 0 | FEMALE | 64 | 5.5 | Ⅲ | 1 | -8.65 |
| 32 | 1713 | 0 | MALE | 60 | 1.4 | Ⅲ | 1 | -8.6 |
| 46 | 1840 | 1 | FEMALE | 59 | 4.3 | Ⅲ | 3 | -8.59 |
| 84 | 2131 | 0 | MALE | 73 | 1.2 | Ⅰ | 4 | -8.56 |
| 44 | 245 | 1 | FEMALE | 78 | 3.3 | Ⅳ | 2 | -8.47 |
| 16 | 782 | 0 | MALE | 59 | 4.4 | Ⅲ | 4 | -8.4 |
| 21 | 150 | 1 | FEMALE | 47 | 5.2 | Ⅱ | 1 | -8.4 |
| 27 | 430 | 0 | FEMALE | 81 | 4.5 | Ⅲ | 2 | -8.16 |
| 66 | 1335 | 1 | FEMALE | 75 | 5.1 | Ⅳ | 3 | -8.14 |
| 30 | 552 | 0 | FEMALE | 48 | 1.5 | Ⅳ | 1 | -7.92 |
| 50 | 1695 | 1 | FEMALE | 72 | 4.3 | Ⅲ | 2 | -7.89 |
| 79 | 1102 | 0 | FEMALE | 79 | 1.6 | Ⅲ | 2 | -7.81 |
| 15 | 1691 | 0 | FEMALE | 55 | 3.2 | Ⅰ | 2 | -7.8 |
| 17 | 791 | 0 | MALE | 80 | 3.5 | Ⅳ | 3 | -7.8 |
| 38 | 2089 | 1 | MALE | 53 | 4.4 | Ⅳ | 1 | -7.42 |
| 18 | 1477 | 1 | MALE | 69 | 1.7 | Ⅲ | 1 | -7.38 |
| 5 | 543 | 0 | FEMALE | 52 | 2.1 | Ⅲ | 1 | -7.37 |
| 26 | 2136 | 1 | FEMALE | 73 | 1.5 | Ⅰ | 3 | -7.35 |
| 24 | 1394 | 0 | MALE | 46 | 1.6 | Ⅲ | 4 | -7.32 |
| 23 | 351 | 0 | FEMALE | 68 | 2.6 | Ⅰ | 2 | -7.27 |
| 25 | 1839 | 1 | MALE | 60 | 3.1 | Ⅰ | 4 | -7.26 |
| 56 | 1276 | 0 | FEMALE | 61 | 1.1 | Ⅱ | 2 | -7.26 |
| 81 | 2099 | 0 | FEMALE | 48 | 1.2 | Ⅰ | 1 | -7.25 |
| 77 | 1807 | 1 | FEMALE | 71 | 1.7 | Ⅰ | 1 | -7.25 |
| 35 | 112 | 0 | MALE | 54 | 1.4 | Ⅳ | 2 | -7.23 |
| 47 | 390 | 1 | MALE | 60 | 1.3 | Ⅱ | 2 | -7.22 |
| 29 | 253 | 0 | FEMALE | 61 | 3.1 | Ⅲ | 2 | -7.14 |
| 12 | 270 | 0 | MALE | 56 | 1.2 | Ⅳ | 2 | -7.13 |
| 78 | 1441 | 1 | FEMALE | 48 | 1.2 | Ⅲ | 3 | -7.05 |
| 9 | 1100 | 0 | MALE | 77 | 2.3 | Ⅰ | 3 | -6.98 |
| 7 | 809 | 0 | MALE | 59 | 1.4 | Ⅱ | 1 | -6.96 |
| 45 | 1268 | 0 | FEMALE | 74 | 1.6 | Ⅰ | 1 | -6.92 |
| 59 | 158 | 0 | MALE | 68 | 1.1 | Ⅱ | 2 | -6.76 |
| 74 | 1382 | 1 | FEMALE | 55 | 1.3 | Ⅳ | 1 | -6.73 |
| 37 | 1096 | 0 | MALE | 65 | 2.2 | Ⅱ | 3 | -6.72 |
| 69 | 570 | 0 | FEMALE | 45 | 1.7 | Ⅱ | 1 | -6.67 |
| 39 | 804 | 0 | MALE | 71 | 2.1 | Ⅰ | 2 | -6.64 |
| 85 | 1565 | 1 | MALE | 34 | 2.1 | Ⅳ | 4 | -6.58 |
| 75 | 1472 | 1 | FEMALE | 62 | 1.3 | Ⅱ | 2 | -6.58 |
| 82 | 2279 | 1 | MALE | 71 | 1.1 | Ⅳ | 2 | -6.56 |
| 41 | 1256 | 0 | MALE | 41 | 2.3 | Ⅱ | 1 | -6.48 |
| 51 | 1184 | 1 | FEMALE | 62 | 3.2 | Ⅱ | 3 | -6.38 |
| 4 | 957 | 0 | MALE | 39 | 0.9 | Ⅳ | 3 | -6.38 |
| 57 | 166 | 1 | FEMALE | 46 | 1.6 | Ⅰ | 1 | -6.32 |
| 48 | 47 | 0 | FEMALE | 68 | 1.2 | Ⅲ | 2 | -6.3 |
| 64 | 864 | 1 | FEMALE | 66 | 2.1 | Ⅱ | 3 | -6.24 |
| 19 | 1585 | 1 | MALE | 53 | 1 | Ⅱ | 2 | -6.18 |
| 2 | 149 | 0 | FEMALE | 61 | 2.3 | Ⅰ | 3 | -6.17 |
| 80 | 1969 | 0 | MALE | 44 | 1.3 | Ⅱ | 1 | -6.13 |
| 52 | 2163 | 0 | FEMALE | 60 | 2.4 | Ⅲ | 2 | -6.12 |
| 68 | 1697 | 1 | MALE | 54 | 1.8 | Ⅲ | 2 | -5.9 |
| 36 | 122 | 1 | MALE | 58 | 2 | Ⅲ | 2 | -5.85 |
| 54 | 694 | 0 | MALE | 68 | 1.9 | Ⅱ | 4 | -5.77 |
| 65 | 322 | 0 | FEMALE | 59 | 1.7 | Ⅲ | 4 | -5.57 |
| 71 | 855 | 1 | FEMALE | 60 | 1.2 | Ⅲ | 2 | -5.36 |
| 28 | 1898 | 0 | FEMALE | 61 | 2.3 | Ⅱ | 3 | -5.01 |
| 55 | 563 | 0 | MALE | 70 | 4.3 | Ⅲ | 4 | -4.54 |
| 63 | 49 | 1 | FEMALE | 48 | 1.5 | Ⅱ | 1 | -4.23 |
| 58 | 1057 | 1 | MALE | 47 | 1.6 | Ⅲ | 1 | -4.13 |
| 20 | 86 | 0 | MALE | 68 | 2.4 | Ⅳ | 3 | -3.12 |

**Table S4. Univariate and multivariate analyses of DICER1-AS1 level and overall survival (TCGA).**

| **Variable** | **Univariate analysis** | | | **Multivariate analysis** | | |
| --- | --- | --- | --- | --- | --- | --- |
|  | **HR** | **95% Cl** | **P** | **HR** | **95% Cl** | ***P*-value** |
| **Overall survival** (n = 171) | |  |  |  |  |  |
| Age (years) |  |  |  |  |  |  |
| ≤60 (n = 55) | 1.246 | 0.788-1.970 | 0.346 |  |  |  |
| >60 (n = 116) |  |  |  |  |  |  |
| Gender |  |  |  |  |  |  |
| Female (n = 78) | 0.855 | 0.565-1.294 | 0.458 |  |  |  |
| Male (n = 93) |  |  |  |  |  |  |
| T stage |  |  |  |  |  |  |
| T1 or T2 (n = 29) | 2.053 | 1.060-3.977 | 0.033 | 1.351 | 0.672-2.722 | 0.400 |
| T3 or T4 (n = 142) |  |  |  |  |  |  |
| N stage |  |  |  |  |  |  |
| N0 or NX (n = 50) | 2.065 | 1.242-3.432 | 0.005 | 1.730 | 1.016-2.931 | 0.044 |
| N1 (n = 121) |  |  |  |  |  |  |
| M stage |  |  |  |  |  |  |
| M0 or MX (n = 167) | 0.985 | 0.241-4.018 | 0.983 |  |  |  |
| M1 (n = 4) |  |  |  |  |  |  |
| G grade |  |  |  |  |  |  |
| G1 or G2 (n = 121) | 1.424 | 0.922-2.200 | 0.111 |  |  |  |
| G3 or G4 (n = 50) |  |  |  |  |  |  |
| DICER1-AS1 |  |  |  |  |  |  |
| (n = 171) | 0.737 | 0.598-0.907 | 0.004 | 0.787 | 0.630-0.982 | 0.034 |

HR, hazard rate; CI, confidence interval.

**Table S5. Correlation between miR-5586-5p expression and clinicopathological features in PAAD patients (TCGA).**

| **Characteristics** | **Low expression**  **n = 86** | **High expression**  **n = 85** | ***P*** |
| --- | --- | --- | --- |
| **Age** |  |  |  |
| ≤60 | 25 (28.7%) | 30 (35.7%) | 0.416 |
| >60 | 62 (71.3%) | 54 (64.3%) |  |
| **Gender** |  |  |  |
| Female | 39 (44.8%) | 39 (46.4%) | 0.955 |
| Male | 48 (55.2%) | 45 (53.6%) |  |
| **T stage** |  |  |  |
| T1 or T2 | 8 (9.2%) | 21 (25.0%) | 0.011 |
| T3 or T4 | 79 (90.8%) | 63 (75.0%) |  |
| **N stage** |  |  |  |
| N0 | 20 (23.0%) | 30 (35.7%) | 0.097 |
| N1 | 67 (77.0%) | 54 (64.3%) |  |
| **M stage** |  |  |  |
| M0 | 85 (97.7%) | 82 (97.6%) | 1.000 |
| M1 | 2 (2.3%) | 2 (2.4%) |  |
| **G grade** |  |  |  |
| G1 or G2 | 60 (69.0%) | 61 (72.6%) | 0.721 |
| G3 or G4 | 27 (31.0%) | 23 (27.4%) |  |
| **TNM stage** |  |  |  |
| I + II | 83 (95.4%) | 80 (95.2%) | 1.000 |
| III + IV | 4 (4.6%) | 4 (4.8%) |  |

**Table S6. Univariate and multivariate analyses of the miR-5586-5p level and overall survival (TCGA).**

| Variable | **Univariate analysis** | | | **Multivariate analysis** | | |
| --- | --- | --- | --- | --- | --- | --- |
|  | HR | 95% Cl | *P* | HR | 95% Cl | *P* |
| **Overall survival** (n = 171) | |  |  |  |  |  |
| Age (years) |  |  |  |  |  |  |
| ≤60 (n = 55) | 1.246 | 0.788-1.970 | 0.346 |  |  |  |
| >60 (n = 116) |  |  |  |  |  |  |
| Gender |  |  |  |  |  |  |
| Female (n = 78) | 0.855 | 0.565-1.294 | 0.458 |  |  |  |
| Male (n = 93) |  |  |  |  |  |  |
| T stage |  |  |  |  |  |  |
| T1 or T2 (n = 29) | 2.053 | 1.060-3.977 | 0.033 | 1.256 | 0.615-2.567 | 0.531 |
| T3 or T4 (n = 142) |  |  |  |  |  |  |
| N stage |  |  |  |  |  |  |
| N0 or NX (n = 50) | 2.065 | 1.242-3.432 | 0.005 | 1.809 | 1.069-3.062 | 0.027 |
| N1 (n = 121) |  |  |  |  |  |  |
| M stage |  |  |  |  |  |  |
| M0 or MX (n = 167) | 0.985 | 0.241-4.018 | 0.983 |  |  |  |
| M1 (n = 4) |  |  |  |  |  |  |
| G grade |  |  |  |  |  |  |
| G1 or G2 (n = 121) | 1.424 | 0.922-2.200 | 0.111 |  |  |  |
| G3 or G4 (n = 50) |  |  |  |  |  |  |
| MiR- |  |  |  |  |  |  |
| (n = 171) | 0.985 | 0.973-0.997 | 0.020 | 0.988 | 0.975-0.988 | 0.069 |

HR, hazard rate; CI, confidence interval.
