# Supplementary material for: A reciprocal feedback between N6-methyladenosine reader YTHDF3 and lncRNA DICER1-AS1 promotes glycolysis of pancreatic cancer through inhibiting maturation of miR-5586-5p
Source: J Exp Clin Cancer Res. 2022 Feb 19;41:69. doi: 10.1186/s13046-022-02285-6 (PMC8857805; doi:10.1186/s13046-022-02285-6)
Supplement: Supplementary file 3 — Additional file 3. Figure S1. DICER1-AS1 is downregulated in pancreaticcancer tissues and negatively correlated with glycolysis pathway. Figure S2. Characteristics of lncRNA DICER1-AS1. Figure S3.Knockdown of DICER1-AS1 promotes glycolysis, proliferationand metastasis of PC cells. Figure S4. The expression correlation between DICER1-AS1and DICER1 and Kaplan-Meier survival curves of DICER1 in tumor datasets. Figure S5. DICER1 is a critical targetfor DICER1-AS1 regulating glycolysis. Figure S6. The expressioncorrelation between YY1 and DICER1 in the CCLE databaseand Kaplan-Meier survival curves of YY1 in tumordatasets. Figure S7. DICER1-AS1 regulates DICER1 transcription byrecruiting YY1 to the promoter of DICER1. Figure S8.miR-5586-5pis an essential target for the DICER1-AS1/DICER1 pathway. Figure S9. miR-5586-5p regulates glycolysis by inducingmRNA degradation of glycolytic genes. [file 13046_2022_2285_MOESM3_ESM.docx]

**
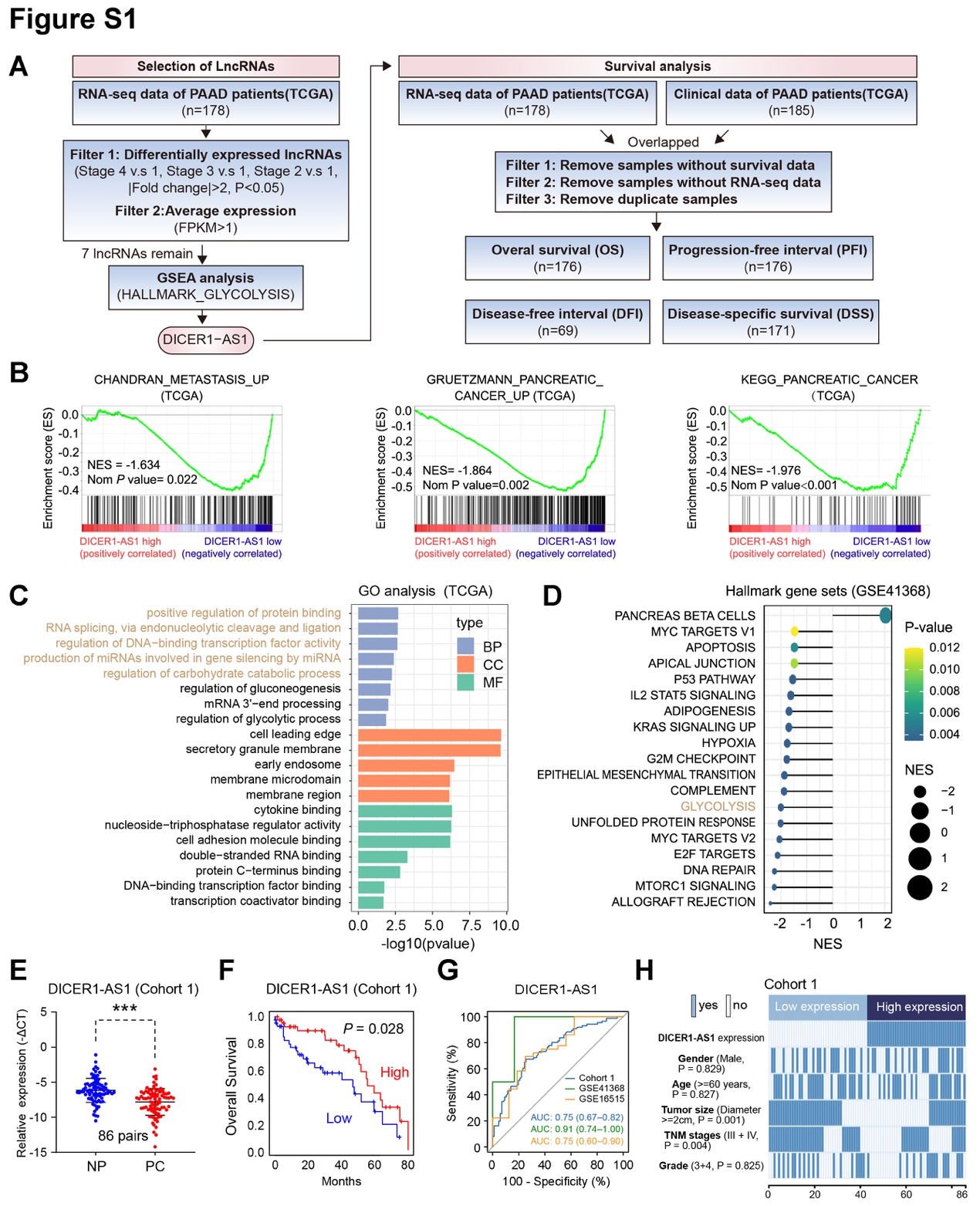
**

**Figure S1.** **DICER1-AS1 is downregulated in pancreatic cancer tissues and negatively correlated with glycolysis pathway.** **A** The flowchart showed the selection of DICER1-AS1 and the data processing of survival data using TCGA database. **B** GSEA analysis of DICER1-AS1-correlated genes. NES, normalized enrichment score. **C** GO analysis of DICER1-AS1-correlated genes based on TCGA database. **D** Bubble plot showing the GSEA analysis of DICER1-AS1-correlated genes in a public microarray dataset (GSE41368). **E** Box plot showing the DICER1-AS1 expression in 86 pairs of pancreatic cancer and adjacent normal tissues using paired t-test. In the plot, the middle line indicates the median, and the whiskers depict the minimum and maximum. **F** Kaplan-Meier curves showing the survival of 86 PC patients with low and high levels of DICER1-AS1 using a log-rank test. **G** ROC analysis of lncRNA DICER1-AS1 in the GEO database (GSE41368, GSE16515) and our specimens. **H** The heatmap indicates the association of different clinical characters (gender, age, tumor size, TNM stage, and grade) with DICER1-AS1 high and low-expression tumors using the χ2 test.


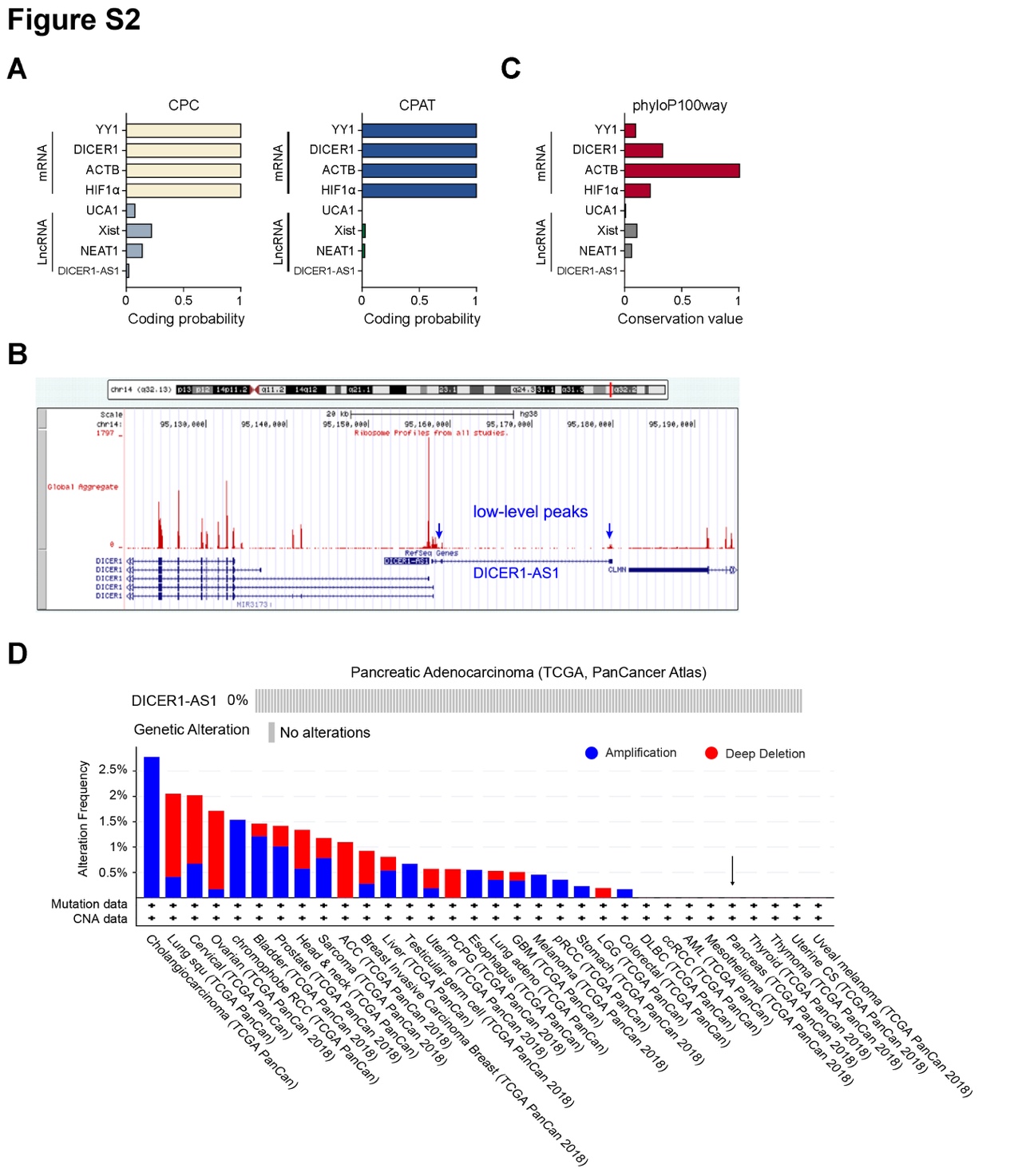


**Figure S2.** **Characteristics of lncRNA DICER1-AS1.** **A** The coding potential of DICER1-AS1 was predicted using web tools of CPAT (http://lilab.research.bcm.edu/cpat/index.php) and CPC (http://cpc2.cbi.pku.edu.cn/). **B** Ribosome profiling data showing the protein-coding potential of DICER1-AS1. **C** The evolutionary conservation of the DICER1-AS1 sequence was calculated using PhyloP across 100 vertebrates. Protein-coding RNAs and lncRNAs were used as control groups. **D** Mining of publicly available datasets derived from cBioPortal for Cancer Genomics (http://www.cbioportal.org/) indicating the frequency of amplification and deletion of the DICER1-AS1 gene in various human cancers.


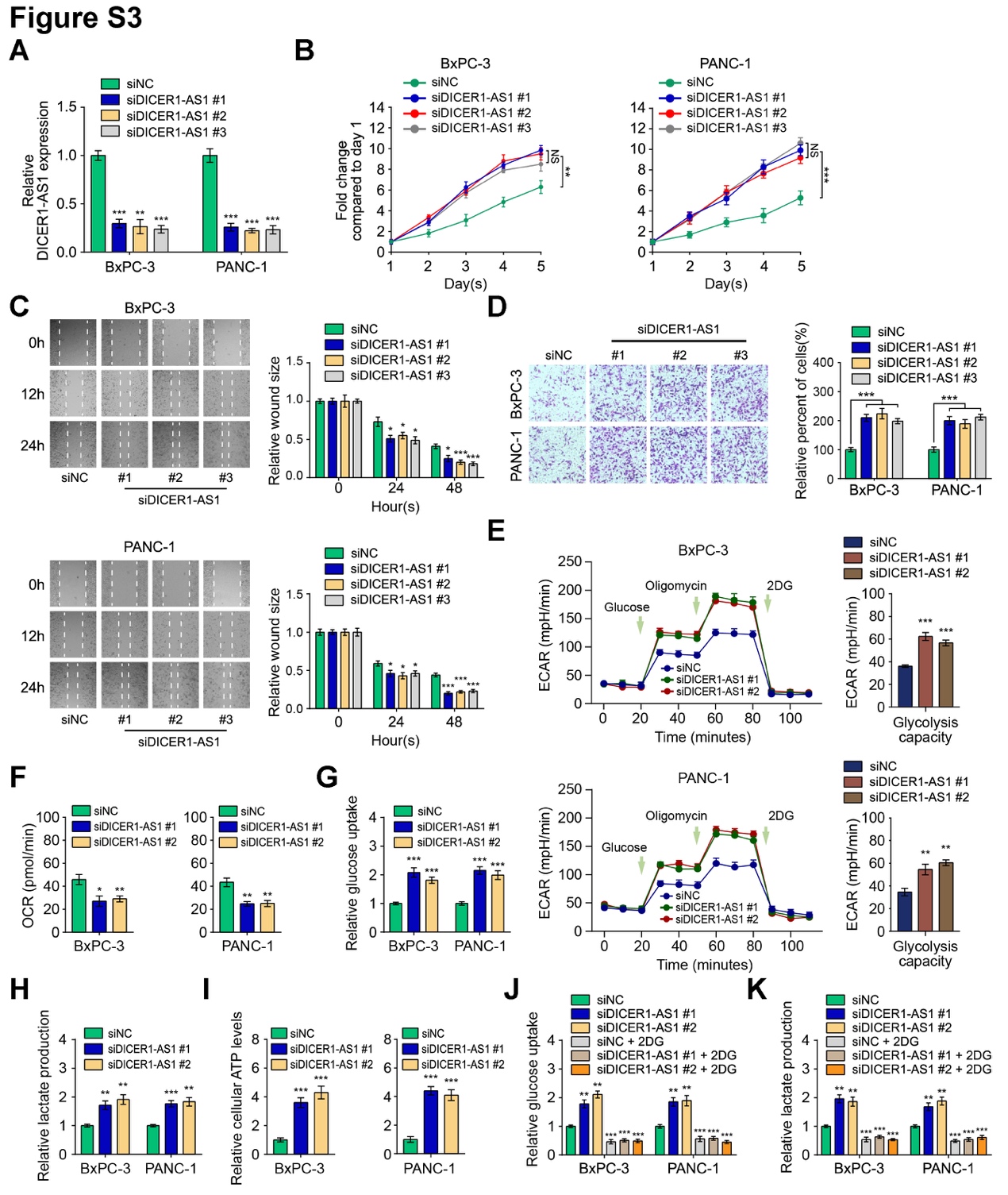


**Figure S3.** **Knockdown of DICER1-AS1 promotes glycolysis, proliferation and metastasis of PC cells. A** The inhibiting efficiency of different siRNA for DICER1-AS1(#1, #2, #3) was evaluated in BxPC-3 and PANC-1 cells, respectively. **B** The proliferative capability was assessed by MTT assay in BxPC-3 and PANC-1 cells transfected with negative control group (siNC) or DICER1-AS1 siRNA groups (#1, #2, #3). **C** Migration ability was assessed by wound healing assay in BxPC-3 and PANC-1 cells transfected with the negative control group (siNC) or DICER1-AS1 siRNA groups (#1, #2, #3). Representative images (left panel) and relative wound sizes were shown (right panel). **D** Transwell assay was applied to assess the invasion ability of those cells transfected indicated above. **E** The change of ECAR levels in BxPC-3 and PANC-1 cells transfected with siNC or siDICER1-AS1 (n=3). The glucose (10 mmol·L^-1^), oligomycin (2 μmol·L^-1^), or 2-deoxyglucose (2-DG, 50 mmol·L^-1^) were treated at indicated points. **F** Seahorse extracellular flux assay showing the oxygen consumption rate (OCR) in BxPC-3 and PANC-1 cells transfected with siNC or siDICER1-AS1 (n=3). **G** The glucose uptake levels in BxPC-3 and PANC-1 cells transfected with siNC or siDICER1-AS1 (n=3). **H** The relative lactic acid levels were detected in BxPC-3 and PANC-1 cells transfected with siNC or siDICER1-AS1 (n=3). **I** The ATP levels in BxPC-3 and PANC-1 cells transfected with siNC or siDICER1-AS1 (n=3). All data were presented as means ± SD of at least three independent experiments. **J-K** The glucose uptake and lactate production were detected in PC cells transfected with control (siNC) or siDICER1-AS1 (#1, #2, #3), and subsequently with 2-DG (10 mmol·L-1, n=5) treatment. Values are significant at *P < 0.05, **P< 0.01 and ***P< 0.001 as indicated.


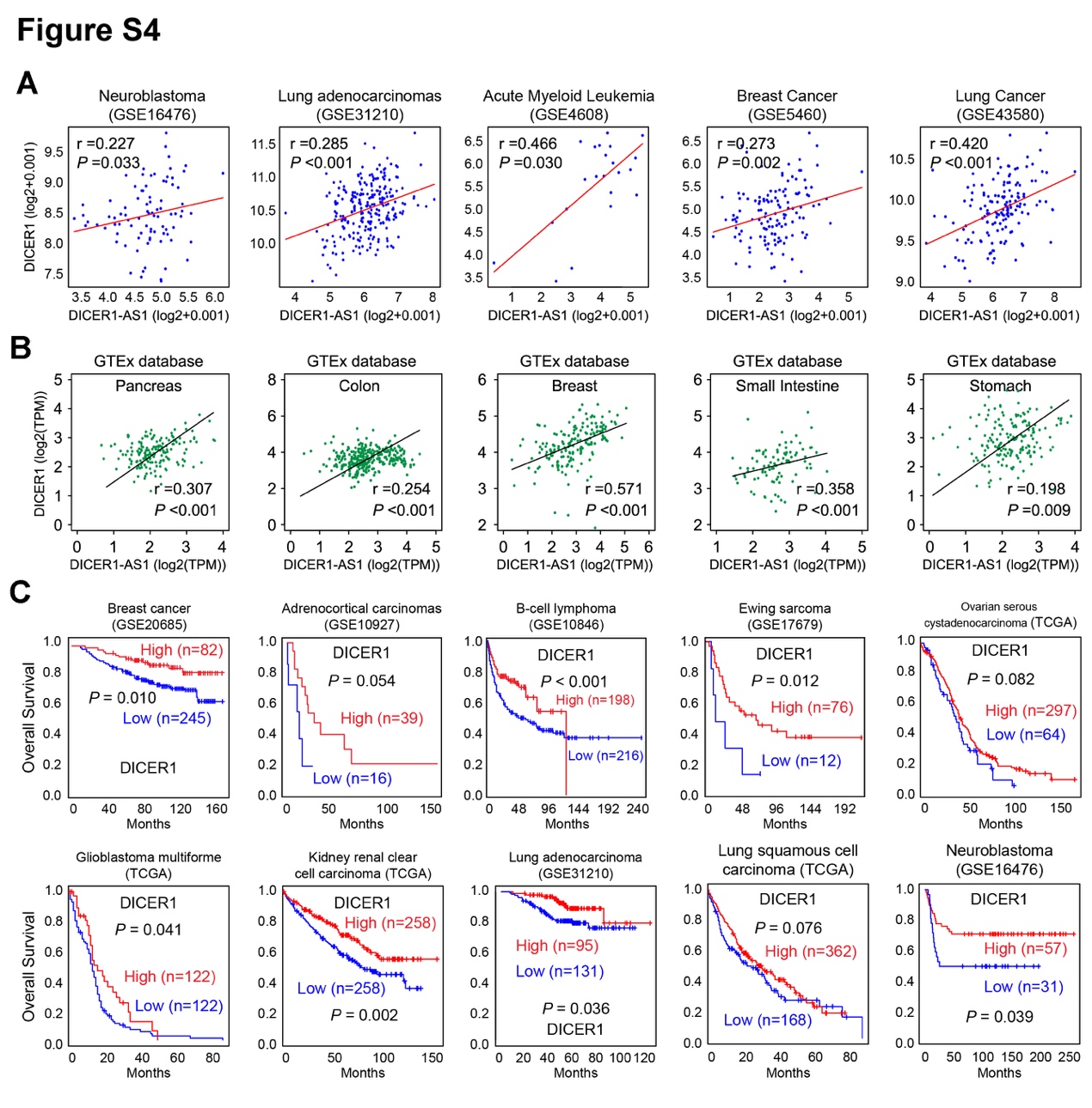


**Figure S4.** **The expression correlation between DICER1-AS1 and DICER1 and Kaplan-Meier survival curves of DICER1 in tumor datasets.** **A** The expression correlation between DICER1-AS1 and DICER1 in GEO datasets of neuroblastoma, lung cancer, acute myeloid leukemia, and breast cancer. **B** The expression correlation between DICER1-AS1 and DICER1 in GTEx datasets of the pancreas, colon breast, small intestine, and stomach. **C** Kaplan-Meier survival analysis of patients with high or low levels of DICER1 in different human tumors derived from GEO database or TCGA database.


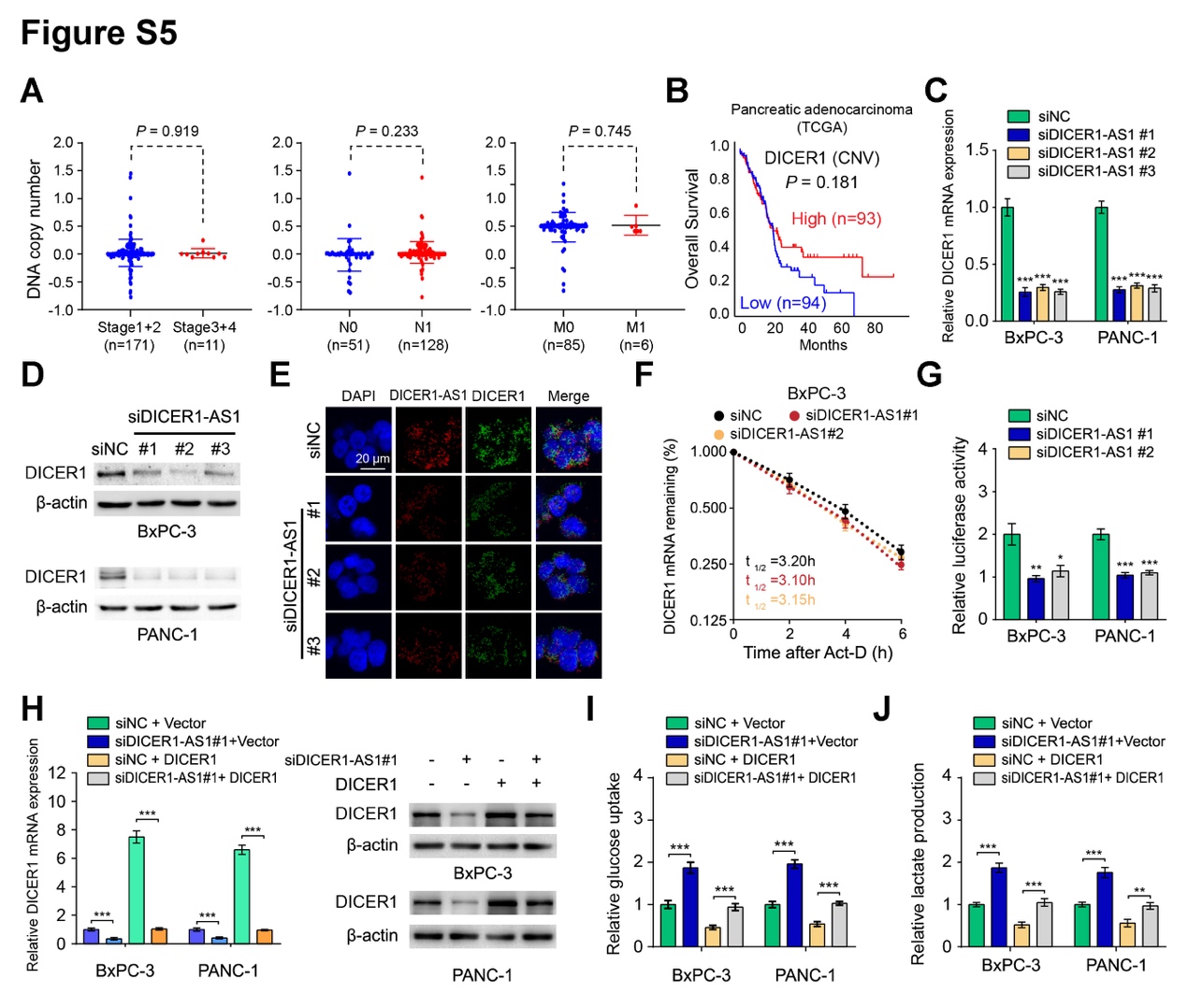


**Figure S5. DICER1 is a critical target for DICER1-AS1 regulating glycolysis.** **A** Box plots showing the copy number of DICER1 gene locus (NC_000014.9, Chromosome 14: 95086228- 95158263, complement) in PC tissues with different status of stages and progression in the TCGA database. **B**. Kaplan-Meier survival analysis of 187 PC patients with high or low DICER1 copy number. **C** Real-time PCR analysis of DICER1 levels in PC cells transfected with siNC or siDICER1-AS1. **D** Western blot assay showing the expression of DICER1 in BxPC-3 and PANC-1 cells transfected with siNC or siDICER1-AS1 (n=3). **E** RNA-FISH and immunofluorescence detection of DICER1-AS1 (red) and DICER1 (green) in DICER1-AS1 knocked down cells (BxPC-3). **F** The DICER1 mRNA half-life (t_1/2_) was detected by real-time PCR in BxPC-3 cells transfected with siNC or siDICER1 (#1, #2). **G** The dual-luciferase assay showing the relative activity of DICER1 promoter in BxPC-3 or PANC-1 cells transfected with siNC or siDICER1-AS1 (#1, #2). **H** Real-time qRT-PCR (left) and western blot (right) showing the change of transcript and protein levels of DICER1 in BxPC-3 and PANC-1 cells transfected with siNC or siDICER1-AS1 and those co-transfected with control or DICER1 plasmids. **I**-**J** The relative glucose uptake and lactic acid production were detected in BxPC-3 or PANC-1 cells transfected with siNC or siDICER1-AS1 and those co-transfected with vector or DICER1 plasmids. All data were presented as means ± SD of at least three independent experiments. Values are significant at *P < 0.05, **P< 0.01 and ***P< 0.001 as indicated.


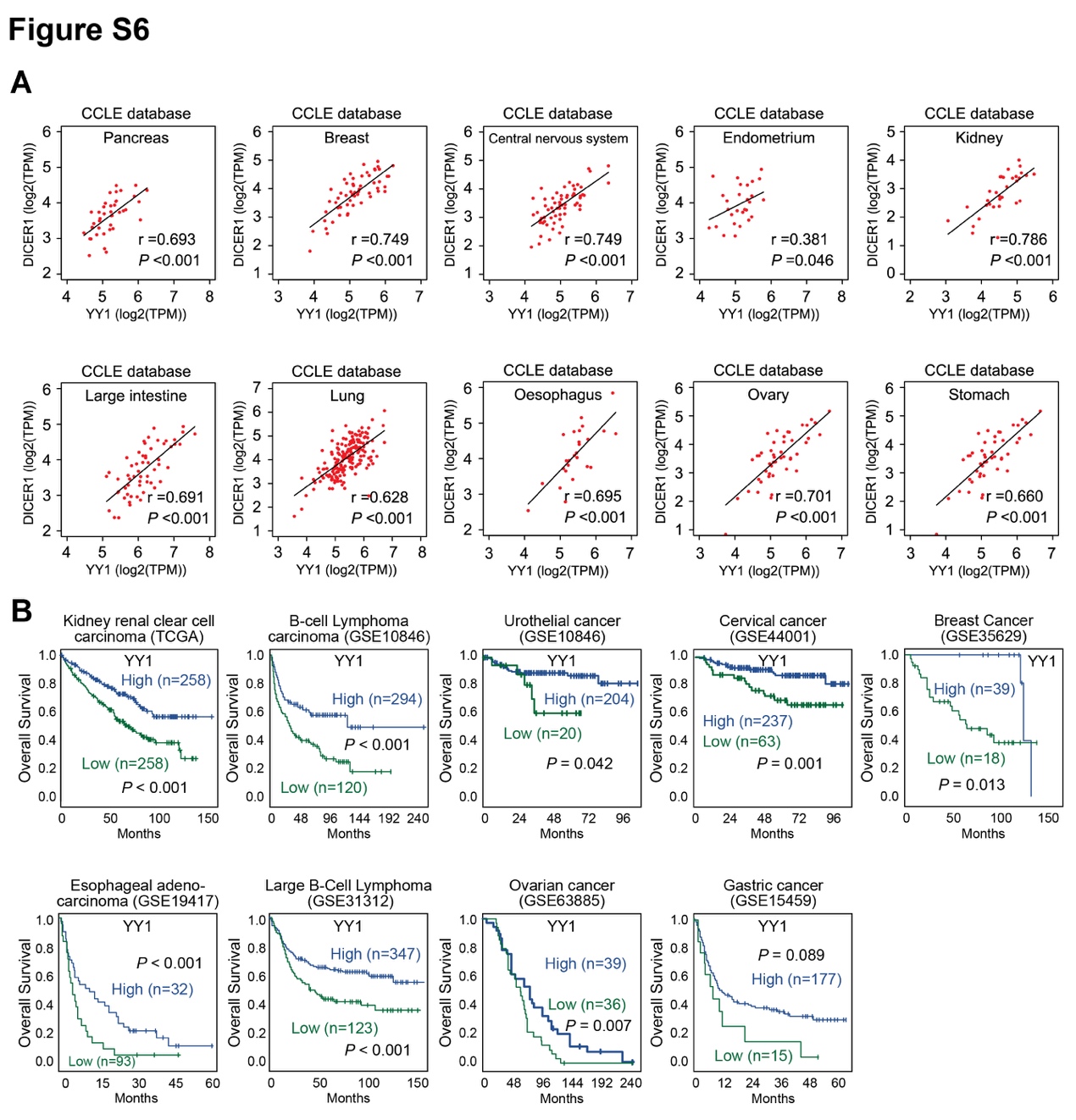


**Figure S6. The** **expression correlation between YY1 and DICER1 in the CCLE database and** **Kaplan-Meier survival curves of YY1 in tumor datasets.** **A** The expression correlation between YY1 and DICER1 in CCLE datasets of the pancreas, breast, central nervous system, endometrium, kidney, large intestine, lung, esophagus, ovary, and stomach. **B** Kaplan-Meier survival analysis of patients with high or low levels of YY1 in different human tumors derived from the GEO database or TCGA database as indicated above.


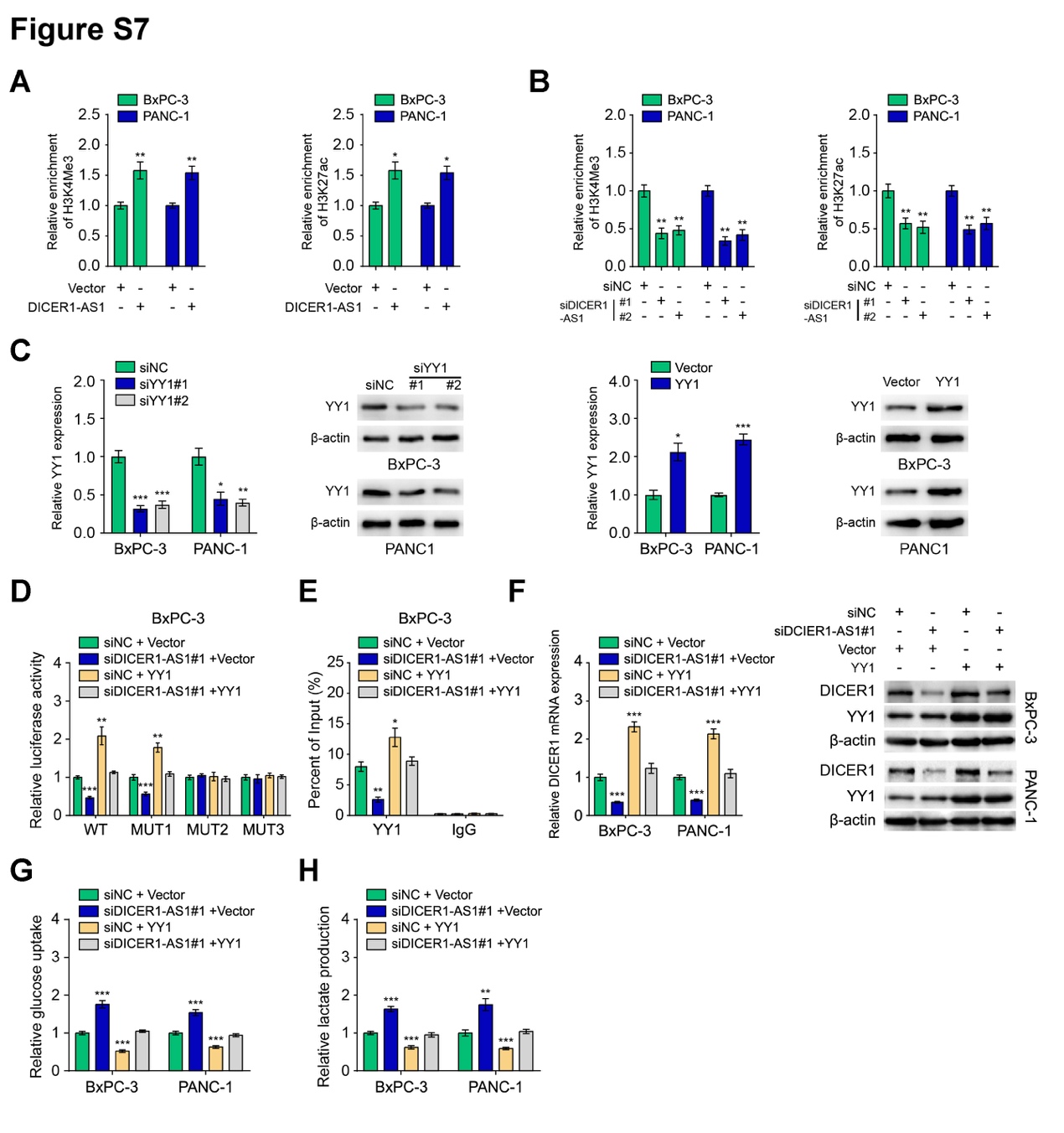


**Figure S7. DICER1-AS1 regulates DICER1 transcription by recruiting YY1 to the promoter of DICER1. A** Relative enrichment of H3K4me3 and H3K27ac to DICER1 promoter was detected via ChIP assay in BxPC-3 and PANC-1 cells transfected with vector control or DICER1-AS1. **B** Relative enrichment of H3K4me3 and H3K27ac to DICER1 promoter was detected via ChIP assay in BxPC-3 and PANC-1 cells transfected with siNC or siDICER1-AS1 (#1, #2). **C** The efficiency of knockdown and overexpression of YY1. **D** The luciferase reporter assays showing the relative activity of DICER1-AS1 and YY1 on the DICER1 promoter in BxPC-3 cells containing WT or MUT reporter vector. **E** Relative enrichment of YY1 to DICER1 promoter was detected via ChIP assay in PC cells transfected with siNC or siDICER1-AS1 and those co-transfected with empty vector or YY1. **F** Real-time qRT-PCR (left panel) and western blot (right panel) showing the changes of transcript and protein levels of DICER1 in BxPC-3 and PANC-1 cells transfected with siNC or siDICER1-AS1 and those co-transfected with vector or YY1 plasmids. **G** The relative glucose uptake was detected in PC cells transfected with siNC or siDICER1-AS1 and those co-transfected with empty vector or YY1. **H** The relative lactic acid production was detected in PC cells transfected with siNC or siDICER1-AS1 and those co-transfected with empty vector or YY1. All data were presented as means ± SD of at least three independent experiments. Values are significant at *P < 0.05, **P< 0.01 and ***P< 0.001 as indicated.


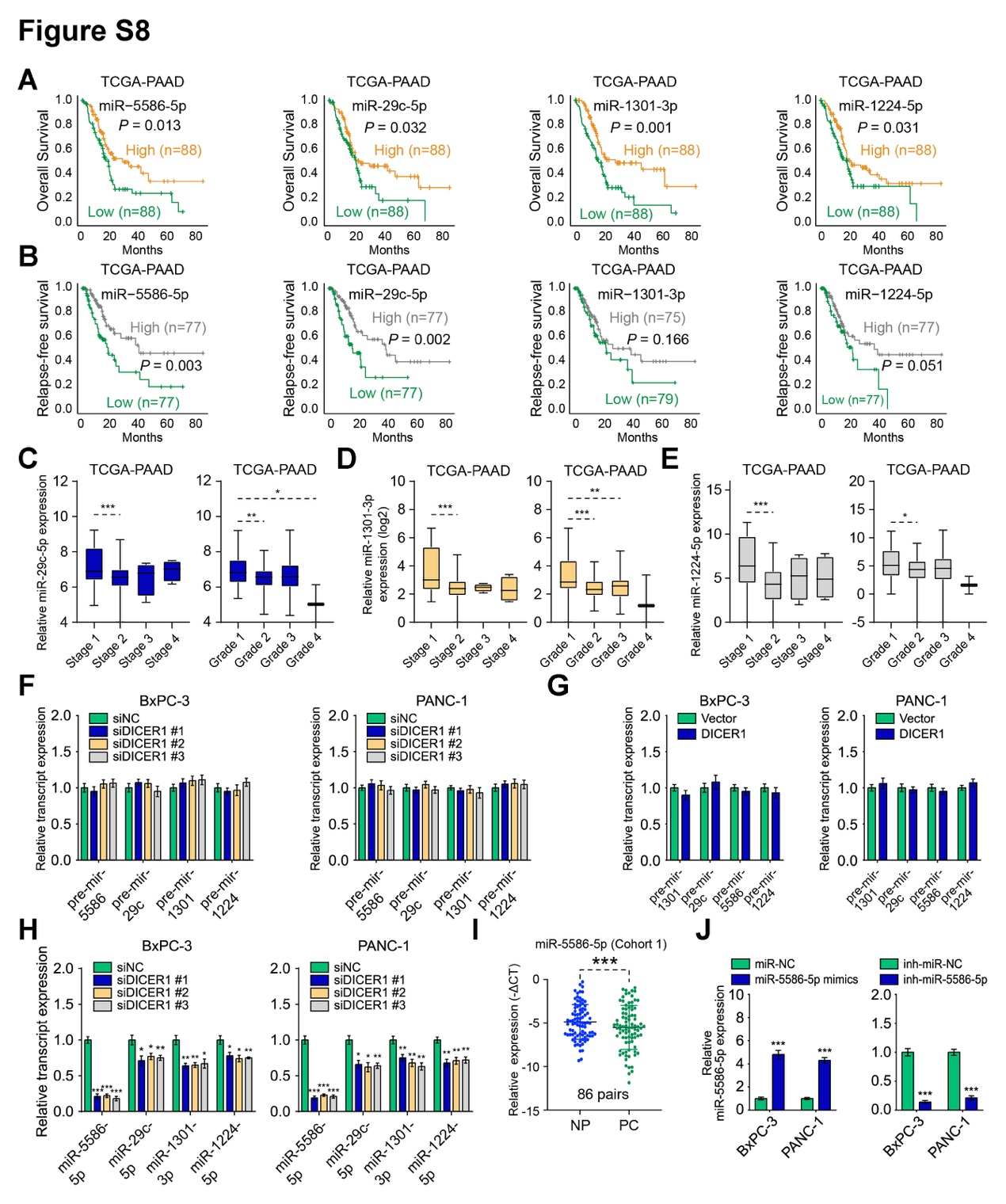


**Figure S8. miR-5586-5p** **is an essential target for the DICER1-AS1/DICER1 pathway.** **A-B** Kaplan-Meier curves showing the overall survival or the relapse-free survival of PC patients with a high or low level of candidate miRNAs, respectively. **C**-**E** Boxplots showing the miR-29c-5p, miR-1301-3p, and miR-1224-5p levels in PC tissues with different statuses of stages and grades. **F-G** Real-time qRT-PCR showing the DICER1 effect on the levels of pre-miRNAs (pre-mir-5586, pre-mir-29c, pre-mir-1301, pre-mir-1224). **H** Real-time qRT-PCR showing the levels of candidate miRNAs in BxPC-3 and PANC-1 cells transfected with siNC or siDICER1 (#1, #2, #3). **I** Box plot showing the miR-5586-5p expression in 86 pairs of pancreatic cancer and adjacent normal tissues using paired t-test. **J** Real-time qRT-PCR analysis showing the miR-5586-5p level in PC cells transfected with miR5586 inhibitors (inh-miR-5586-5p) or mimics (miR-5586-5p-mimics) and corresponding control oligos (inh-miR-NC: inhibitor control, miR-NC: mimics control). All data were presented as means ± SD of at least three independent experiments. Values are significant at *P < 0.05, **P< 0.01 and ***P< 0.001 as indicated.


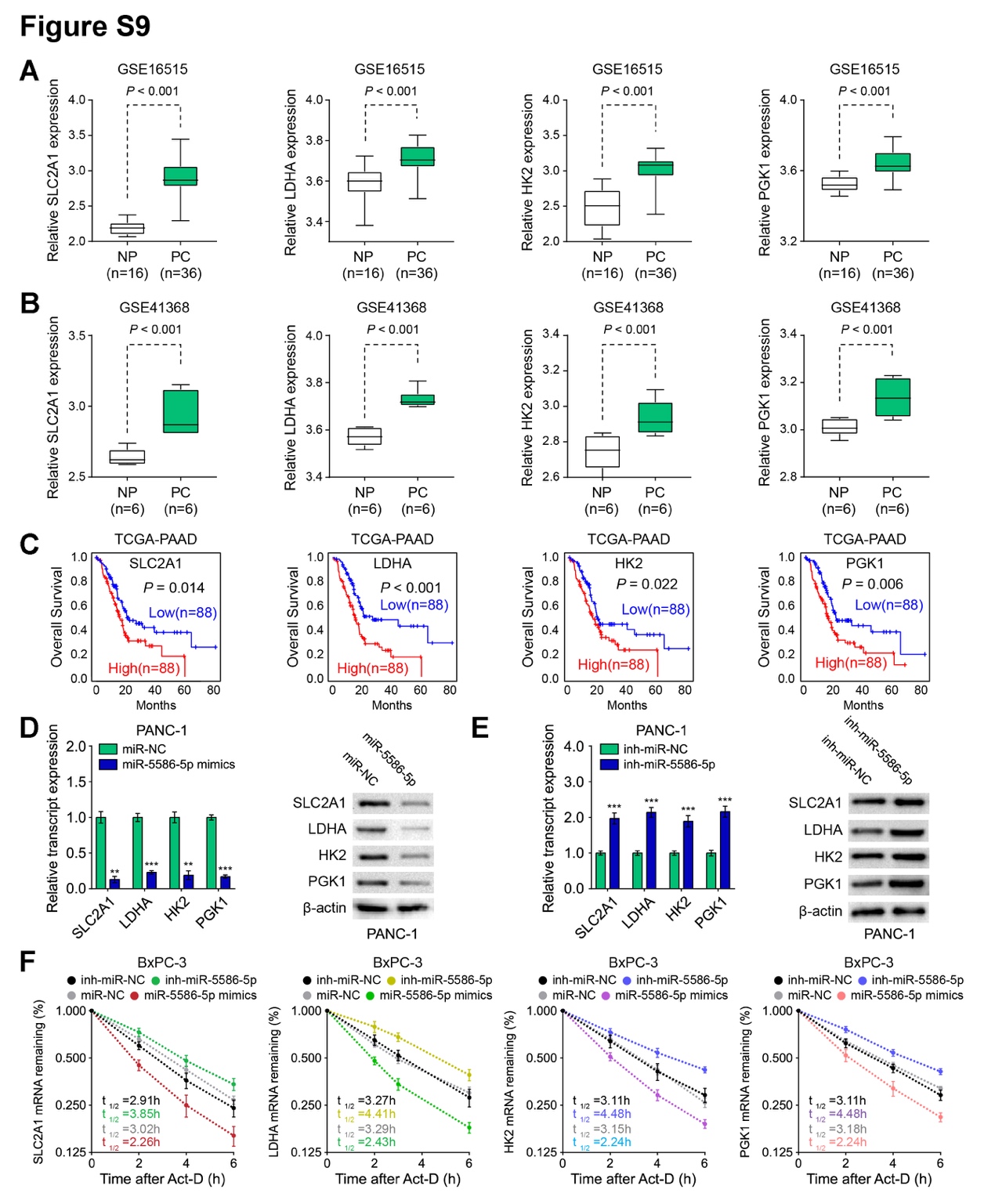


**Figure S9.** **miR-5586-5p regulates glycolysis by inducing mRNA degradation of glycolytic genes.** **A**-**B** Boxplots showing the relative levels of candidate glycolytic genes (SLC2A1, HK2, LDHA, PGK1) in the public GEO database (GSE16515, GSE41368). **C** Kaplan-Meier survival curves of candidate glycolytic genes (SLC2A1, HK2, LDHA, PGK1) in the TCGA database. A log-rank test was used in the survival curves. **D-E** Real-time qRT-PCR (left panel) and western blot (right panel) showing the effects of miR-5586-5p mimics and inhibitors on the levels of glycolytic genes (SLC2A1, HK2, PGK1, LDHA) in PANC-1 cells. **F** The influence of miR-5586-5p mimics and inhibitors on the mRNA half-life (t1/2) time of candidate mRNAs of glycolytic genes in BxPC-3 cells. All data were presented as means ± SD of at least three independent experiments. Values are significant at *P < 0.05, **P< 0.01 and ***P< 0.001 as indicated.


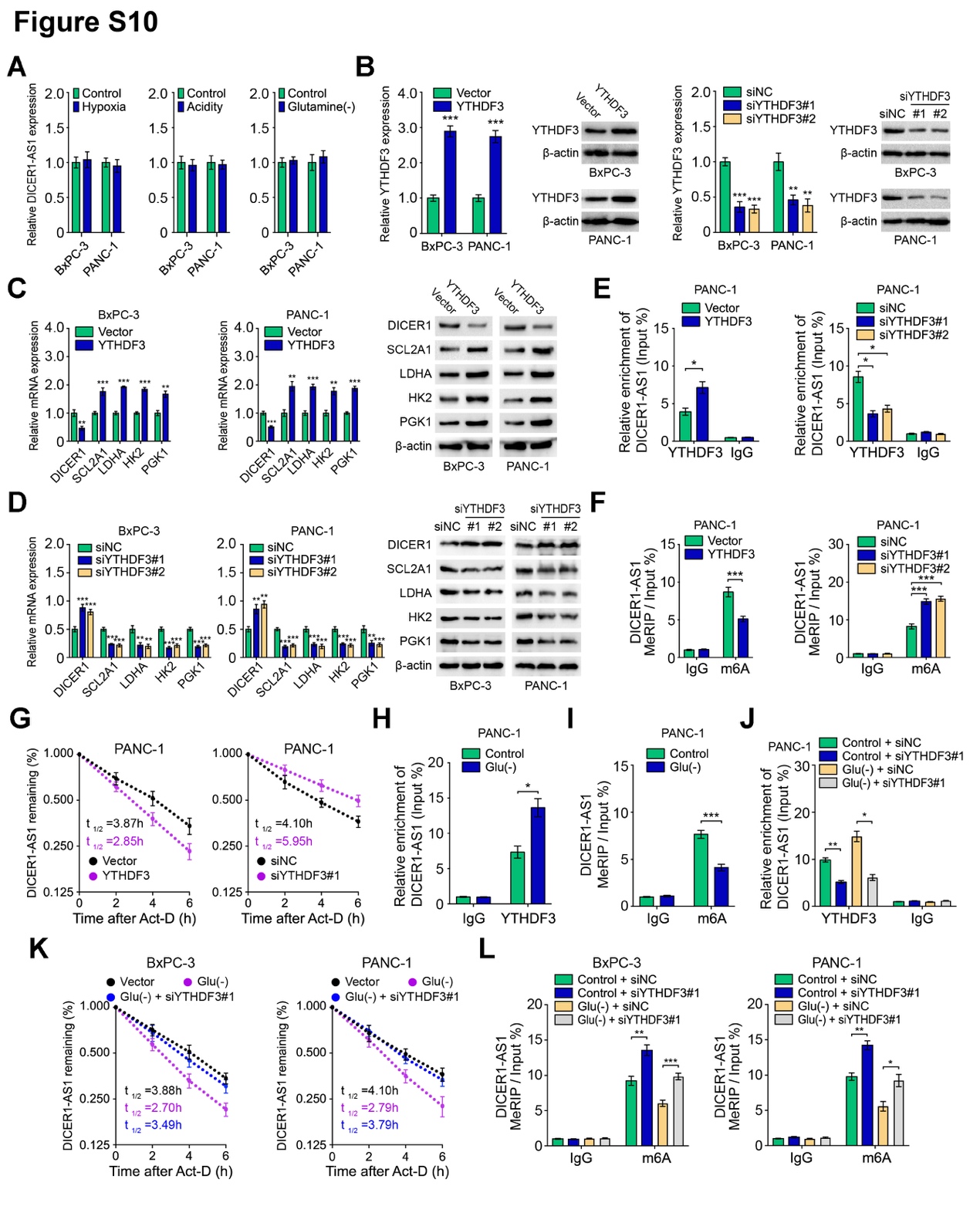


**Figure S1****0.** **m6A reader YTHDF3 decreases DICER1-AS1 stability of PC cells in response to glucose deprivation.** **A** The DICER1-AS1 level was detected by qRT-PCR in BxPC-3 and PANC-1 cells under different environments including hypoxia, acidity, or glutamine starvation. **B** The efficiency of knockdown and overexpression of YTHDF3. **C-D** The mRNA and protein levels of DICER1 and glycolytic genes in PC cells influenced by YTHDF3. **E** RIP assay showed the effects of YTHDF3 on the enrichment of DICER1-AS1 with YTHDF3 protein in PANC-1 cells. **F** The MeRIP‐qPCR showed the effects of YTHDF3 on the enrichment of m6A on DICER1-AS1 in PANC-1 cells. **G** The effects of YTHDF3 on the RNA half-life (t1/2) time of DICER1-AS1 in actinomycin D-treated PANC-1 cells. **H** RIP assay showed the enrichment of DICER1-AS1 on YTHDF3 protein in PANC-1 cells cultured in a normal or glucose-free medium for 48h. **I** The MeRIP‐qPCR showed the m6A modification level of DICER1-AS1 in PANC-1 cells cultured in a normal or glucose-free medium for 48h. **J** RIP assay showed the enrichment of DICER1-AS1 on YTHDF3 protein in PANC-1 cells transfected with siNC or siYTHDF3#1 in the normal or glucose-free medium. **K** The effects of siYTHDF3#1 and glucose deprivation on the RNA half-life (t1/2) time of DICER1-AS1 in actinomycin D-treated PC cells. **L** The MeRIP‐qPCR showed the m6A modification level of DICER1-AS1 in PC cells transfected with siNC or siYTHDF3 under normal or glucose deprivation for 48h. All data were presented as means ± SD of at least three independent experiments. Values are significant at *P < 0.05, **P< 0.01 and ***P< 0.001 as indicated.


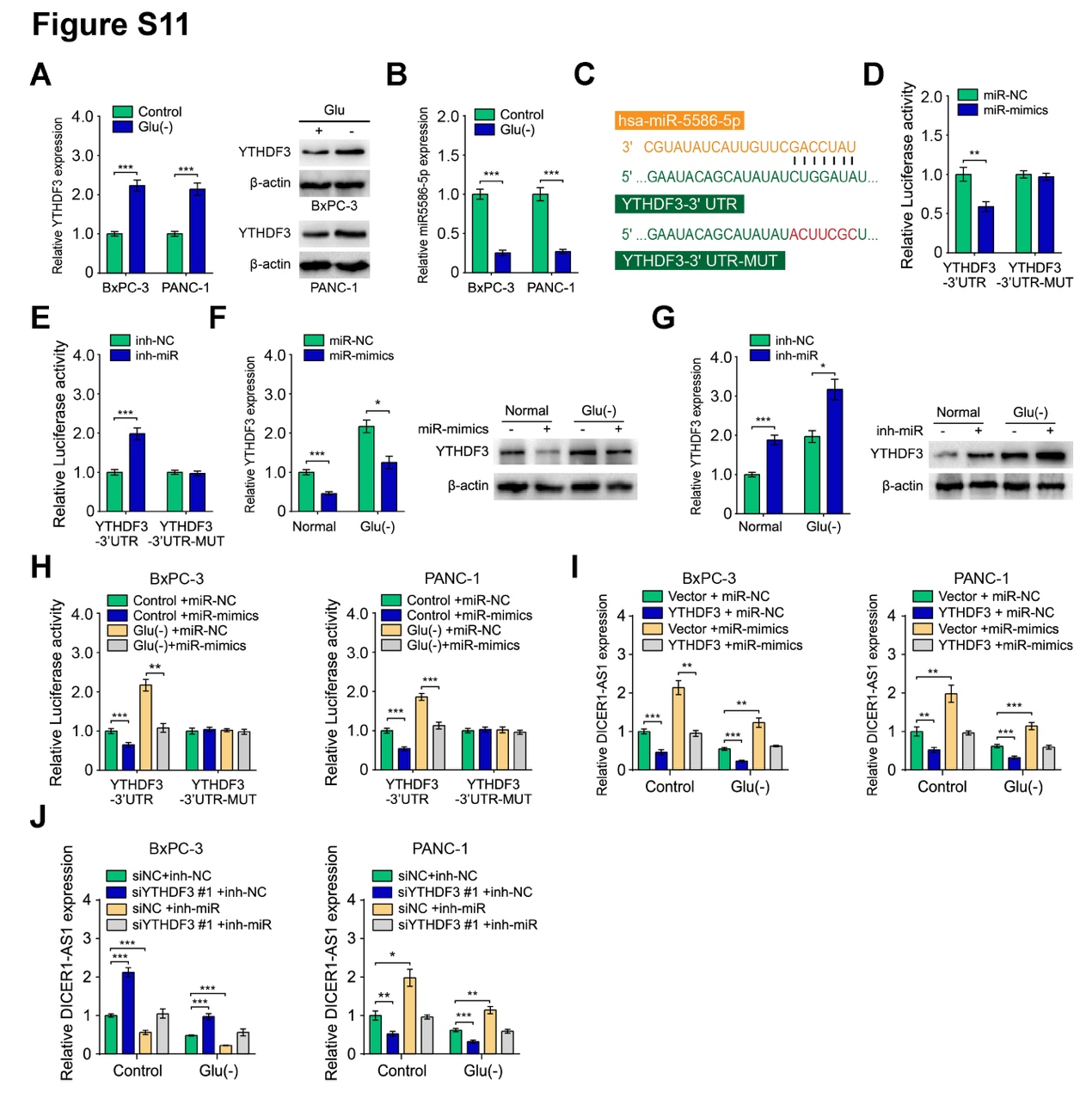


**Figure S11.** **YHDF3 is a reciprocal target for DICER1-AS1 via miR-5586-5p-induced inhibition. A** Real-time qRT-PCR (left panel) and western blot (right panel) showing the effects of the glucose-free environment on the level of YTHDF3 in BxPC-3 and PANC-1 cells. **B** Real-time qRT-PCR showing the effect of the glucose-free environment on the level of miR-5586-5p in BxPC-3 and PANC-1 cells. **C** Schematic illustration showing the YTHDF3 3’UTR fragment containing wild-type or mutated miR-5586-5p binding site predicted by TargetScan database (<http://www.targetscan.org/vert_72/>). WT: wild type; MUT: mutant type. **D**-**E** The luciferase reporter assays showing the effects of miR-5586-5p mimics and inhibitors on the reporter activity of YTHDF3 3’UTR (WT, MUT) in BxPC-3 and PANC-1 cells in the normal or glutamine-free medium. **F**-**G** After transfected with miR-5586-5p mimics or inhibitors, the expression of YTHDF3 was evaluated by qRT-PCR (left panel) and western blot (right panel) in BxPC-3 cells. **H** The YTHDF3 3’UTR reporter activities (WT or MUT) were detected by luciferase reporter assays in PC cells transfected with the miR-5586-5p mimics in the normal or glucose-free medium. **I-J** Real-time qRT-PCR showing the effect of YTHDF3 and miR-5586-5p on DICER1-AS1 level in BxPC-3 and PANC-1 cells. All data were presented as means ± SD of at least three independent experiments. Values are significant at *P < 0.05, **P< 0.01 and ***P< 0.001 as indicated.


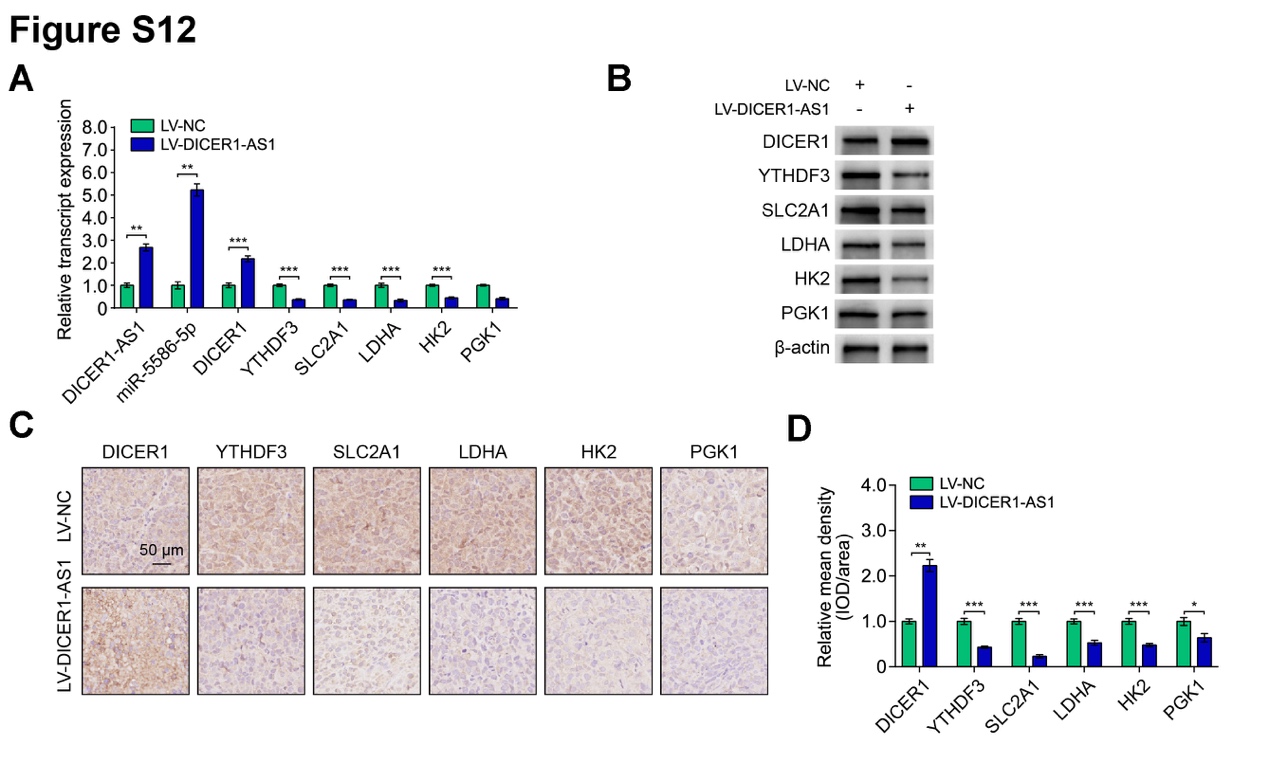


**Figure S12. The YTHDF3/DICER1-AS1/DICER1/miR-5586-5p axis is pivotal for glycolysis and tumorigenesis of PC.** **A**-**B** Real-time qRT-PCR and western blot showing the expression of DICER1-AS1, DICER1, miR-5586-5p, YTHDF3, and glycolytic genes within subcutaneous xenograft tumors in nude mice that received an intravenous injection of LV-NC or LV-DICER1-AS1. **C-D** Immunohistochemical staining and relative mean density (integrated option density/area) of indicated protein. All data were presented as means ± SD of at least three independent experiments. Values are significant at *P < 0.05, **P< 0.01 and ***P< 0.001 as indicated.
